# Supplementary material for: Cracking the black box of deep sequence-based protein–protein interaction prediction
Source: Brief Bioinform. 2024 Mar 5;25(2):bbae076. doi: 10.1093/bib/bbae076 (PMC10939362; doi:10.1093/bib/bbae076)
Supplement: Supplementary_Material_bbae076 [file supplementary_material_bbae076.pdf]

Cracking the black box of deep sequence-based protein-protein interaction prediction - Supplementary Material -

Judith Bernett[1,*], David B. Blumenthal[2,†], and Markus List[1,†]

[1]Data Science in Systems Biology, TUM School of Life Sciences, Technical University of Munich, Freising, Germany
[2]Biomedical Network Science Lab, Department Artificial Intelligence in Biomedical Engineering, Friedrich-Alexander-Universität Erlangen-Nürnberg, Erlangen, Germany
[*]Corresponding author: judith.bernett@tum.de
[†]These authors contributed equally.

Keywords: PPI prediction, Data Leakage, Deep Learning

Tested methods

The results of an extensive literature screening for high-performing PPI prediction methods can be found in Supplemental Table S1. Only twelve of 32 reviewed publications made their code available (13 methods; Richoux et al.[1] proposed two). Eight methods were written in Python (PIPR[2], DeepFE[3], Richoux-FC, Richoux-LSTM[1], D-SCRIPT[4], Topsy-Turyv[5], PRoBERTa[6], and TransformerGO[7]), two in Matlab (You et al.[8], Ding et al.[9]), one in Lua (Hashemifar et al.[10]), and two in C++ (Hamp and Rost[11], SPRINT[12]). We excluded the methods that did not only use sequences as input (Hashemifar et al.[10], Hamp and Rost[11], TransformerGO[7]) and focused on DL methods with high reported accuracies, which we managed to reproduce with reasonable effort. Additionally, we included the SPRINT method as a baseline comparison since it only relies on sequence similarity for its predictions. As further similarity-based baselines, we included simple baseline ML methods (random forest, SVM), which we ran on three different encodings for the amino acid sequences (PCA, MDS, node2vec). We included two node label classification algorithms, which we ran on the line graphs of the PPI networks (harmonic function, global and local consistency) to test how much can be predicted using topology alone. Overall, we hence tested seven published methods and eight baseline ML models.

PIPR. PIPR[2] encodes the two input sequences using pre-trained embeddings. These embeddings take co-occurrence similarities of amino acids and physicochemical properties into account. PIPR feeds the embeddings to a deep siamese network: the embeddings are input to two residual recurrent CNN units (convolutional layer, max pooling, bidirectional GRU with residual shortcuts, global average pooling) with shared weights. The outputs are then combined using element-wise multiplication. Finally, the network predicts binary PPIs by training a multi-layer perceptron with leaky ReLU (final activation function: softmax, loss function: binary cross-entropy, optimizer: adam with AMSgrad, learning rate= 0.001, epsilon = $1e^{-6}$). For our tests, we fixed the hyperparameters according to the results of the original publication (number of epochs: 50, batch size: 256, dimension of the convolutional and GRU layers: 25, sequence embedding cropped at position: 2000).

DeepFE. DeepFE[3] pre-trained a Word2vec/Skip-gram model (size: 20, window: 4), using all Swissprot protein sequences (from 2018) as input. The model treats every amino acid as a word and, therefore, a sequence as a sentence. PPI candidate sequences are then represented using the Word2vec embeddings and are fed to a deep siamese network consisting of four units (dense layer with ReLU, batch normalization, dropout layer) with decreasing dimensionality (2048, 1024, 512, and 128). The outputs are then concatenated and put through another unit with dimensionality 8. PPI prediction is made at the final two-dimensional layer with a soft-max activation function. A stochastic gradient descent optimizer is used (learning rate: 0.01, momentum: 0.9, decay: 0.001). For our tests, we fixed the hyperparameters according to the results of the original publication (number of epochs: 45, batch size: 256, maximum protein length fed to the model: 850).

Richoux-FC and Richoux-LSTM. Richoux et al.[1] represent all sequences using one-hot encoding and present two DL models: a fully connected model and a recurrent LSTM model, which we refer to as Richoux-FC and Richoux-LSTM. Richoux-FC first flattens both sequence inputs and passes them separately through two units consisting of a dense layer (dimensionality: 20, ReLU) and a batch normalization layer. The two outputs are concatenated and passed to another unit (dense layer, dimensionality: 20, ReLU). The final layer (dimensionality: 1) predicts the PPI using a sigmoid activation function. Richoux-LSTM first extracts features from the sequence via three units (convolution, pooling, batch normalization) and a final LSTM layer. The parameters are shared for these layers for the two input sequences. Then, the outputs are concatenated and passed to a dense layer (ReLU) with another batch normalization. Finally, the LSTM model predicts the PPIs using a sigmoid activation function on the final one-dimensional dense layer. Both models use the Adam optimizer with an initial learning rate of 0.001, which reduces by 10% if the loss plateaus for 5 epochs until a minimum of 0.0008. For our tests, we fixed the hyperparameters according to the results of the original publication (number of epochs for Richoux-FC: 25, number of epochs for Richoux-LSTM: 100, batch size: 256, maximum sequence length: 1166).

D-SCRIPT and Topsy-Turvy Before running the D-SCRIPT model, sequences are embedded using a pre-trained language model by Bepler and Berger[13], which transforms a sequence of length n to a matrix of dimension $n \times 6165$. In our case, we embedded the yeast and the human proteome separately. The two embeddings of a PPI between proteins of length n and m are passed separately through a projection module with shared weights, which reduces the dimensionality to $n \times 100$ and $m \times 100$, respectively. This module consists of a dense layer (dimensionality: $n \times 100$ and $m \times 100$, respectively, with ReLu activation), followed by a dropout layer. The outputs are combined by concatenating their element-wise differences and Hadamard products and used as input for the residue contact module. A dense layer (dimensionality: 50), followed by a batch normalization layer and a ReLU activation, produces a tensor of dimensionality $n \times m \times 50$. D-SCRIPT passes the matrix to a convolutional layer (width: 7), followed by a batch normalization layer and a sigmoid activation function to produce a contact prediction matrix $\hat{C} \in [0,1]^{n \times m}$. The interaction prediction module transforms this matrix into an interaction probability by subjecting it to a max-pooling operation (size: 9), a custom global pooling operation, and a custom logistic activation function. The loss function minimized during training is a weighted sum of the binary cross-entropy loss (prediction) and a contact-map magnitude loss. We fixed all hyperparameters to the ones from the publication, i.e., training for 10 epochs with a batch size of 25 and an Adam optimizer with a learning rate of 0.001.

Topsy-Turvy computes a topology score, the GLIDE score, and the D-SCRIPT prediction probability for each PPI. The GLIDE score combines a local, neighborhood-based metric (common weighted normalized score) and a global metric (diffusion state distance) to capture the network surrounding the two protein nodes, regardless of whether they are close (local metric) or not (global metric). The score is binarized at a cutoff of 92.5. A GLIDE score prediction loss extends the part of the D-SCRIPT loss responsible for interaction probability with a relative importance of 0.2. The GLIDE score prediction

loss is the binary cross-entropy loss between the D-SCRIPT prediction and the binarized GLIDE score. Hyperparameters were fixed as specified in the publication, i.e., training for 10 epochs with a batch size of 25 and an Adam optimizer with a learning rate of 0.001.

SPRINT. Instead of finding hidden patterns via DL, SPRINT[12] entirely relies on the hypothesis that a protein pair that is pairwise similar to an interacting protein pair is more likely to interact. Therefore, we can use it as a baseline model to see how well a dataset can be predicted if we only use sequence similarities (Explanation 3). SPRINT searches for similar subsequences between the candidate protein pair and the database of known interactions by using an approach very similar to BLAST's hit-and-extend approach. Instead of having consecutive initial seeds of a certain length like BLAST, SPRINT also allows for gapped initial seeds to increase the number of hits. Using all hits, SPRINT calculates similarity scores and sorts the output decreasingly by the scores where a higher score represents a higher probability for interaction.

Baseline ML models

Similarity-based models We implemented additional baseline models to compare the performance of DL methods against classical ML. Note that the idea behind implementing the baseline models is not to show that classical ML models suffice for the PPI prediction task but to explain the phenomenal accuracies reported for sequence-based DL models. Because of this, we designed our baseline models such that they can only learn from sequence similarities and node degrees: the similarities are the only input features and the node degrees can be learnt implicitly during training.

As models, we trained a random forest (sklearn 1.0.2 RandomForestClassifier, 100 decision trees, six parallel jobs, a prediction greater than 0.5 is interpreted as interaction) and an SVM (sklearn SVC, RBF kernel, maximum number of iterations: 1000, a prediction > 0.5 is interpreted as interaction). We adopted these hyperparameters from the sklearn defaults; no tuning was done.

Proteins were represented using a similarity vector where each entry is the pairwise similarity score (bitscore) to every other protein in the human or yeast proteome (defined by Swissprot). A PPI was encoded as the concatenation of the two embeddings. An all-against-all similarity matrix for human and yeast was computed by the team of SIMAP2[14], yielding a 6718×6718 matrix for yeast and a $20\,353 \times 20\,353$ matrix for human. Since this dimensionality is too high, we reduced it via three approaches: PCA, MDS, and node2vec, each returning 128 dimensions.

Theoretically, preprocessing the whole dataset before splitting it into train and test is considered data leakage. In this case, it is acceptable since we consider the whole universe (proteome). Introducing data leakage via joint preprocessing usually means that we have information available during testing that we would not have in a real scenario (e.g., divergence from the mean of the dataset). However, the dimensionality reductions are computed on the similarity matrix of all known human proteins; therefore, we do not introduce any dataset-specific biases. Given a new PPI, we would already possess the embeddings of its individual proteins.

PCA and MDS yielded matrices of dimensions 6718×128 and $20\,353 \times 128$ for the yeast and human proteomes. For the computation of the node2vec encoding, we built a weighted similarity network from the similarity matrix such that an edge between two proteins exists if and only if their bitscore is positive. Self-loops were excluded. Since SIMAP2 uses cutoffs, some of the proteins had no edges. Because the input to node2vec is just an edge list, the resulting node2vec embeddings were smaller, resulting in matrices of dimensions 6194×128 and $20\,210 \times 128$ for the yeast and human proteomes. Altogether, we hence tested three different embeddings as input to two ML models, yielding six baseline models.

Topology-based models Both models are node label classification algorithms implemented in NetworkX 2.8. Because the PPI labels are edge labels, we first converted all PPI networks to line graphs for this task (i.e., all edges become nodes, and all nodes become edges, Supplementary Figure S1).

Zhu et al.[15] formulate the problem of assigning labels to unlabelled nodes in a partially labeled network (our line graph) in terms of a Gaussian random field on the graph. The solution is given by the minimum harmonic energy function, which is unique and can be calculated explicitly using matrix operations. In NetworkX 2.8, the solution is calculated iteratively. On an unweighted graph, in the first iteration, node label probabilities are calculated by looking at the labels of neighboring, labeled nodes. In the next iteration, these probabilities are updated according to the label probabilities of the neighboring nodes and the node labels. This procedure is repeated n times, in our case, $n = 30$.

Zhou et al.[16] slightly modified the method presented by Zhu et al., causing information to be spread symmetrically. They also introduce a parameter specifying the relative amount of information a node receives from its neighbors (here, $\alpha = 0.99$) and the initial labels $(1 - \alpha)$. A final difference is that, in contrast to the harmonic function, the label probabilities also influence the initial node labels, so outliers can potentially be corrected. Like the harmonic function, the local and global consistency algorithm is iterated n times, here $n = 30$.

Table S1: Literature overview. Datasets: ∗ GUO (yeast), ○ PAN (human), † MARTIN (H. pylori), • Cross-species DIP dataset (E. coli: E, H. sapiens: H, C. elegans: C, M. musculus: M, H. pylori: P, D. melanogaster: D), ⋄ HUANG (human), ⊗ DU (yeast). Citations: Number of Citations reported on Google Scholar on 25.05.23. **Code available.**

Paper	Method	Input	Reported accuracies	Reported on	Citations
1. Guo et al. (2008)[17]	AC encoding + SVM	Sequence only.	0.87∗, other own datasets: 0.58-0.86	2/5 random test	667
2. Guo et al. (2010)[18]	SVM	Sequence only.	0.89∗, 0.91○, 0.93 E/ 0.90 D/ 0.978 C •, other: 0.94	2/5 random test	69
3. Pan et al. (2010)[19]	CT encoding + hierarchical LDA-RF	Sequence only.	0.98○	Mean 5CV	176
4. Sun et al. (2017)[20]	AC encoding + stacked autoencoder	Sequence only.	0.97○, 0.5†, 0.96 E/ 0.98 D/ 0.97 C •, other: 0.9-0.99	ca. 1/10 random test	317
5. **Chen et al. (2019, PIPR)[2]**	Embeddings, siamese RCNN, MLP	Sequence only.	0.97∗	Mean 5CV	157
6. Wang et al. (2019)[21]	PSSM, CNN with FSRF	PSSM (PSI-BLAST)	0.98∗, 0.89†	Mean 5CV	77
7. Xu et al. (2020)[22]	Feature extraction: physicochem. graph energy, contact graph energy, dipeptide composition. PCA, WSRC classifier	Sequence only.	1.0∗, 0.97†, 0.99⋄	Mean 5CV	9
8. Wang et al. (2017)[23]	LCTD encoding, NN	Sequence only.	0.87†, 0.95 E/ 0.93 C/ 0.94 H/ 0.93 M •, 0.93⊗	Mean 5CV	39
9. **You et al. (2015)[8]**	MLD feature representation + RF	Sequence only.	0.95∗, 0.88†, 0.89 E/ 0.88 C/ 0.94 H/ 0.92 M/ 0.91 P •	Mean 5CV	160
10. Hu et al. (2015)[24]	frequently occurring variable-length sequence segments, patterns sign. compared to background, weight, probability for interaction	Sequence only.	0.62 ∗ (AUC), 0.68 ○ (AUC)	Mean 5CV	23
11. Du et al. (2017)[25]	Feature extraction, Siamese DNN	Sequence only.	0.86†, 0.92 E/ 0.95 C/ 0.94 H/ 0.91 M •, 0.98⋄, 0.93⊗, other: 0.79-0.91	1/4 random test	172

Table S1: Literature overview. Datasets: ∗ GUO (yeast), ○ PAN (human), † MARTIN (H. pylori), • Cross-species DIP dataset (E. coli: E, H. sapiens: H, C. elegans: C, M. musculus: M, H. pylori: P, D. melanogaster: D), ⋄ HUANG (human), ⊗ DU (yeast). Citations: Number of Citations reported on Google Scholar on 25.05.23. **Code available.**

12. **Yao et al. (2019, DeepFE)**[3]	Res2Vec encoding + siamese network	Sequence only.	0.95∗, 1 E/ 1 C/ 1 H/ 1 M •, 0.99⋄, other: 0.73-0.94	2/5 random test	51
13. Jha & Saha (2020)[26]	ResNet50 on 3D data, LSTM + Encoder/Decoder on sequence AC/CT embedding	Sequence + 3D structure	0.97○, 0.94⊗	Mean 3CV	15
14. Saha et al. (2014)[27]	Ensemble Learning: SVM, RF, decision tree, naive Bayes	Overlap of GO graphs, interacting domains, paralogous verification method.	own datasets 0.68-0.91	Mean 10CV	54
15. Chen et al. (2019)[28]	AC encoding, GO LCA clustering, functional similarity graph, Resniks measure on GO + Ensemble learner: RF, naive Bayes, NN, KNN. SVM for classification	Sequence, GO terms, topological features	0.84∗, 0.98 H/ 0.95 E/ 0.98 D/ 0.99 C •, 0.92⊗, other: 0.78-0.94	Mean 10CV	52
16. Zhao et al. (2020)[29]	sequence, GO: Bert + BIGRU, Inception CNN, Attention, GAP, GMP	Sequence + GO terms	0.97∗, 0.90⊗, other: 0.88-0.96	Mean 10CV	11
17. **Hashemifar et al. (2018)**[10]	PSSM, siamese-like CNN, random projection module	PSSM (PSI-BLAST)	0.95∗, 0.96 H/ 0.97 E/ 0.96 C/ 0.96 M •	Mean 10CV and 5CV	248
18. Maetschke et al. (2021)[30]	Different encodings of GO terms + RF	GO terms.	0.9∗, other: 0.73-0.93	Mean 10CV	85
19. Shen et al. (2007)[31]	CT encoding, SVM	Sequence only.	own dataset 0.84	ca. 1/100, repeated 5 times	1028
20. Mahapatra et al. (2020)[32]	AAC encoding + PCA/PSO, SVM	Sequence only.	own dataset: 0.99	2/5 random test	3
21. Wang et al. (2018)[33]	PSSM, PCA, rotation forest	PSSM (PSI-BLAST)	0.97∗, 0.88†, 0.92 H/ 1.0 E/ 0.91 C/ 0.91 M •	Mean 5CV	35

Table S1: Literature overview. Datasets: ∗ GUO (yeast), ○ PAN (human), † MARTIN (H. pylori), • Cross-species DIP dataset (E. coli: E, H. sapiens: H, C. elegans: C, M. musculus: M, H. pylori: P, D. melanogaster: D), ⋄ HUANG (human), ⊗ DU (yeast). Citations: Number of Citations reported on Google Scholar on 25.05.23. **Code available.**

22. **Hamp & Rost (2015)**[11]	Evolutionary profile kernel, k-mers + SVM	Sequence + Predict-Protein.	own datasets: 0.67-0.87	Mean 10CV	107
23. **Li & Ilie (SPRINT, 2017)**[12]	spaced seeds, hit-and-extend, scores for similar subsequences	Sequence only.	own datasets: 0.74-0.93, other: 0.61-0.82	Mean 10 CV	65
24. **Ieremie et al. (TransformerGO, 2022)**[7]	GO-sets, graph embeddings (node2vec), Transformer	GO terms.	other: 0.91-0.97	2/5 random test	11
25. **Ding et al. (2016)**[9]	Multivariate MI feature representation, Moreau-Broto Autocorrelation + RF	Sequence only.	0.95∗, 0.88 †, 0.94 H/ 0.93 E/ 0.92 C/ 0.96 M •, 0.98 ⋄	Mean 10CV	139
26. **Richoux et al. (FC, LSTM, 2019)**[1]	Fully connected and recurrent deep models	Sequence only.	own datasets: 0.76-0.9	ca. 12% random and ca. 0.6% strict test set	24
27. **Sledzieski et al. (D-SCRIPT, 2021)**[4]	Pre-trained protein-wise embedding, fully connected projection module, convolutional contact module	Sequence only.	own datasets: 0.405-0.580 auPR	Mean 5CV, cross-species test	39
28.**Singh et al. (Topsy-Turvy, 2022)**[5]	D-SCRIPT + GLIDE score for network topology	Sequence only.	own datasets: 0.533-0.824 auPR	Mean CV, cross-species test	6
29. **Nambiar et al. (PRoBERTa, 2020)**[6]	Task-agnostic transformer for protein sequence encoding, fine-tuning for PPI prediction (concatenated sequence) and family classification	Sequence only.	own datasets: 0.79-0.89	10% random test	77
30. Pazos et al. (Mirrortree, 2001)[34]	MSA of protein orthologs for both proteins, correlate evolutionary distance matrices	Sequence, MSAs.	None, just correlations	-	648

Table S1: Literature overview. Datasets: ∗ GUO (yeast), ○ PAN (human), † MARTIN (H. pylori), • Cross-species DIP dataset (E. coli: E, H. sapiens: H, C. elegans: C, M. musculus: M, H. pylori: P, D. melanogaster: D), ⋄ HUANG (human), ⊗ DU (yeast). Citations: Number of Citations reported on Google Scholar on 25.05.23. **Code available.**

31. Ochoa et al. (p-Mirrortree, 2015)[35]	Computes p-values for mirrortree scores from a background distribution	Sequence, MSAs.	own datasets: ca. 0.15 PPV, ca. 0.14 F1	whole dataset	27
32. Humphreys et al. (2021)[36]	Pipeline: MSAs of yeast orthologs, contact probabilities with RoseTTAFold, refinement with AlphaFold	Sequence, MSAs.	Comparison to PDB, detailed analysis of single structures	-	231

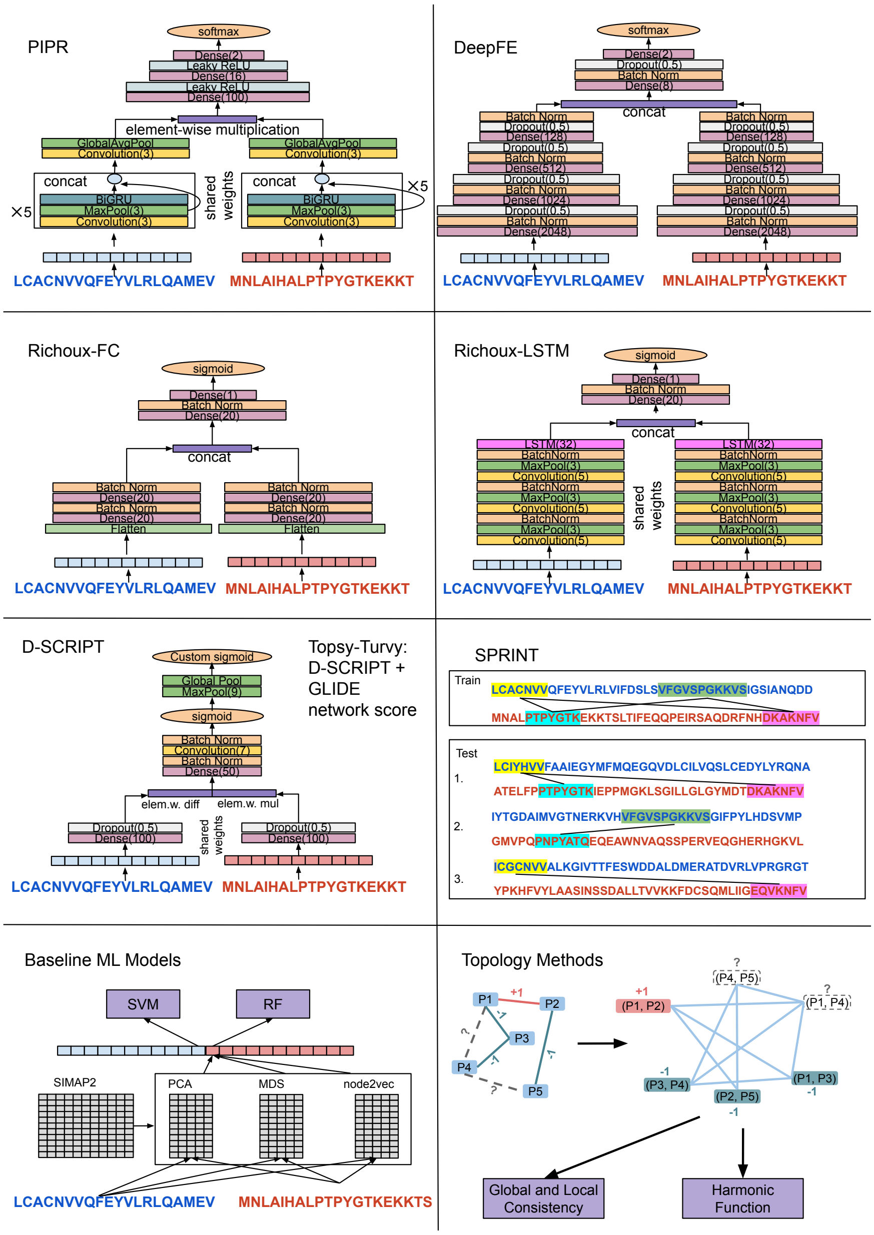

Figure S1: Overview of all tested methods. The DL methods all propose siamese network architectures where the sequences are first processed individually (shared weights for PIPR, Richoux-LSTM, and D-SCRIPT). The output is then combined and processed by fully connected layers. Predictions are made by a final softmax (PIPR, DeepFE) or sigmoid (Richoux-FC, Richoux-LSTM, D-SCRIPT) activation function. SPRINT examines candidate PPIs for subsequences similar to subsequences of known interacting proteins. Scores are calculated from pairs of subsequences, indicated by black lines. Our baseline models embed proteins using a dimensionality-reduced (PCA / MDS / node2vec) sequence-similarity vector (bitscores computed by SIMAP2). The embeddings are concatenated for a PPI and fed to either a random forest or a support vector machine. The topology methods work on the line graphs of the PPI networks and infer the missing node labels of the test set.

Dataset	Unique Whole Data	Overlap Whole Data/Train	Overlap Train/Test	% of Test Set
HUANG	2,571	2,354	1,086	83.3%
HUANG$_{LR}$	2,053	1,883	831	83.0%
GUO	2,497	2,494	2,089	99.9%
GUO$_{LR}$	2,209	2,207	1,776	99.9%
DU	4,417	4,258	2,969	94.9%
DU$_{LR}$	3,977	3,813	2,566	94.0%
PAN	9,122	8,631	5,335	91.6%
PAN$_{LR}$	7,587	7,166	4,293	91.1%
D-SCRIPT UNBAL.	14,227	14,227	13,937	100%
D-SCRIPT UNBAL.$_{LR}$	14,214	14,214	13,924	100%
RICHOUX-REGULAR	17,245	16,847	5,398	93.1%
RICHOUX-REGULAR$_{LR}$	16,640	16,261	5,190	93.2%
RICHOUX-STRICT	15,831	15,512	291	47.7%
RICHOUX-STRICT$_{LR}$	15,318	15,042	256	48.1%

Table S2: Number of unique proteins occurring in the original datasets (from positive and negative interactions), number of unique proteins occurring in the training set, overlap of unique proteins occurring in both the train and the test set, and proportion of the overlap w.r.t the test set. Numbers are displayed for the normal and length-restricted (LR) datasets.

Dataset	Unique Whole Data	Overlap Whole Data/Train	Overlap Train/Test	% of Test set
HUANG	2,268	1,979	1,023	78.0%
HUANG$_{LR}$	1,835	1,616	790	78.3%
GUO	2,493	2,488	2,075	99.8%
GUO$_{LR}$	2,206	2,197	1,746	99.5%
DU	4,198	3,945	2,886	91.9%
DU$_{LR}$	3,783	3,540	2,476	91.1%
PAN	8,412	7,692	5,019	87.5%
PAN$_{LR}$	7,030	6,424	4,036	86.9%
D-SCRIPT UNBAL.	14,227	14,227	13,937	100%
D-SCRIPT UNBAL.$_{LR}$	14,214	14,214	13,923	100%
RICHOUX-REGULAR	17,222	16,749	5,326	91.8%
RICHOUX-REGULAR$_{LR}$	16,634	16,185	5,124	91.9%
RICHOUX-STRICT	15,798	15,477	289	47.4%
RICHOUX-STRICT$_{LR}$	15,282	15,008	252	47.9%

Table S3: Number of unique proteins occurring in the rewired datasets (from positive and negative interactions), number of unique proteins occurring in the training set, overlap of unique proteins occurring in both the train and the test set, and proportion of the overlap w.r.t the test set. Numbers are displayed for the normal and length-restricted (LR) datasets.

Dataset	Unique Whole Data	Whole Data ∩ Train	Train ∩ Test	% of Test Set
HUANG $INTER \rightarrow INTRA_0$	2,224	1,833	736	65.3%
HUANG$_{LR}$ $INTER \rightarrow INTRA_0$	1,728	1,442	542	65.5%
HUANG $INTER \rightarrow INTRA_1$	2,182	1,833	570	62.0%
HUANG$_{LR}$ $INTER \rightarrow INTRA_1$	1,774	1,442	479	59.1%
HUANG $INTRA_0 \rightarrow INTRA_1$	2,046	1,127	0	0.0%
HUANG$_{LR}$ $INTRA_0 \rightarrow INTRA_1$	1,639	828	0	0.0%
GUO $INTER \rightarrow INTRA_0$	2,371	2,034	1,091	76.4%
GUO$_{LR}$ $INTER \rightarrow INTRA_0$	2,052	1,752	915	75.3%
GUO $INTER \rightarrow INTRA_1$	2,159	2,034	690	84.7%
GUO$_{LR}$ $INTER \rightarrow INTRA_1$	1,877	1,752	611	83.0%
GUO $INTRA_0 \rightarrow INTRA_1$	2,243	1,428	0	0.0%
GUO$_{LR}$ $INTRA_0 \rightarrow INTRA_1$	1,951	1,215	0	0.0%
DU $INTER \rightarrow INTRA_0$	4,211	3,844	1,950	84.2%
DU$_{LR}$ $INTER \rightarrow INTRA_0$	3,753	3,403	1,668	82.7%
DU $INTER \rightarrow INTRA_1$	4,053	3,844	1,373	86.8%
DU$_{LR}$ $INTER \rightarrow INTRA_1$	3,632	3,403	1,239	84.4%
DU $INTRA_0 \rightarrow INTRA_1$	3,899	2,317	0	0.0%
DU$_{LR}$ $INTRA_0 \rightarrow INTRA_1$	3,486	2,018	0	0.0%
PAN $INTER \rightarrow INTRA_0$	8,146	6,545	3,271	67.1%
PAN$_{LR}$ $INTER \rightarrow INTRA_0$	6,652	5,370	2,610	67.1%
PAN $INTER \rightarrow INTRA_1$	7,513	6,545	2,459	71.8%
PAN$_{LR}$ $INTER \rightarrow INTRA_1$	6,362	5,370	1,990	66.7%
PAN $INTRA_0 \rightarrow INTRA_1$	8,299	4,872	0	0.0%
PAN $INTRA_0 \rightarrow INTRA_1$	6,874	3,892	0	0.0%
D-SCRIPT UNBAL. $INTER \rightarrow INTRA_0$	17,659	14,213	7,035	67.1%
D-SCRIPT UNBAL.$_{LR}$ $INTER \rightarrow INTRA_0$	16,015	14,200	7,023	79.5%
D-SCRIPT UNBAL. $INTER \rightarrow INTRA_1$	14,213	14,213	7,178	100%
D-SCRIPT UNBAL.$_{LR}$ $INTER \rightarrow INTRA_1$	14,200	14,200	7,177	100%
D-SCRIPT UNBAL. $INTRA_0 \rightarrow INTRA_1$	17,659	10,481	0	0.0%
D-SCRIPT UNBAL.$_{LR}$ $INTRA_0 \rightarrow INTRA_1$	16,015	8,838	0	0.0%
RICHOUX-UNIPROT $INTER \rightarrow INTRA_0$	16,789	15,439	7,765	85.2%
RICHOUX-UNIPROT$_{LR}$ $INTER \rightarrow INTRA_0$	15,985	14,646	7,287	84.5%
RICHOUX-UNIPROT $INTER \rightarrow INTRA_1$	16,415	15,439	5,766	85.5%
RICHOUX-UNIPROT$_{LR}$ $INTER \rightarrow INTRA_1$	15,645	14,646	5,459	84.5%
RICHOUX-UNIPROT $INTRA_0 \rightarrow INTRA_1$	15,857	9,115	0	0.0%
RICHOUX-UNIPROT$_{LR}$ $INTRA_0 \rightarrow INTRA_1$	15,084	8,626	0	0.0%

Table S4: Number of unique proteins occurring in the partitioned datasets (from positive and negative interactions), number of unique proteins occurring in the training set, overlap of unique proteins occurring in both the train and the test set, and proportion of the overlap w.r.t the test set. Numbers are displayed for the normal and length-restricted (LR) datasets.

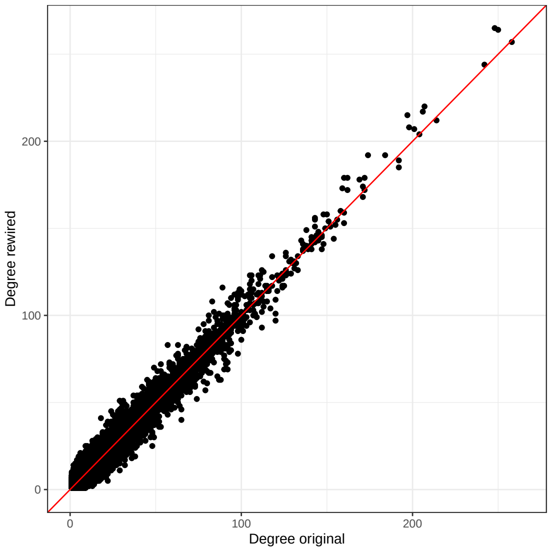

Figure S2: Node degrees of the proteins in the original vs. rewired networks. This serves merely as a sanity check to see that indeed, the rewiring preserved the node degrees for all proteins in expectation.

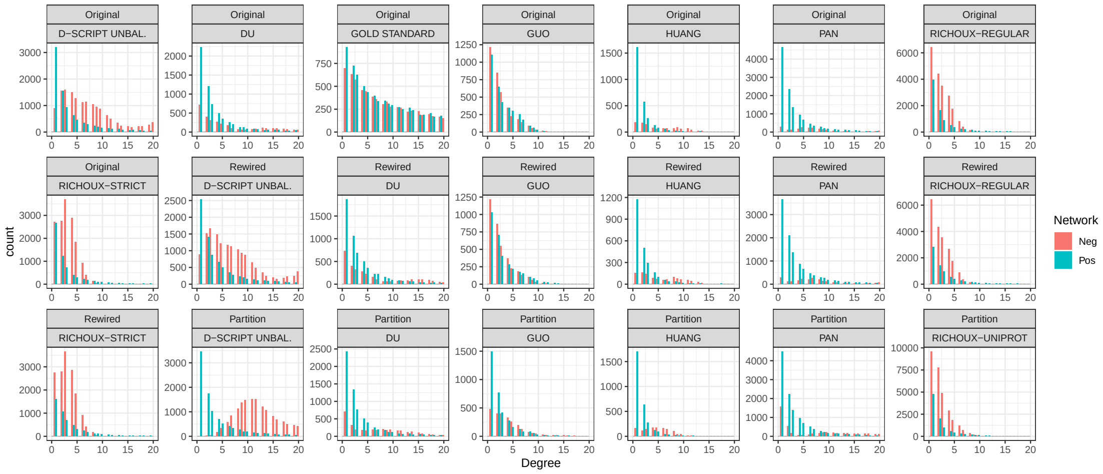

Figure S3: Comparison of the node degrees of the positive vs. negative networks for all datasets. The distributions follow the same trend for all datasets except for HUANG and PAN. For these two, negative PPIs were sampled uniformly while the positive PPI distribution follows the power law.

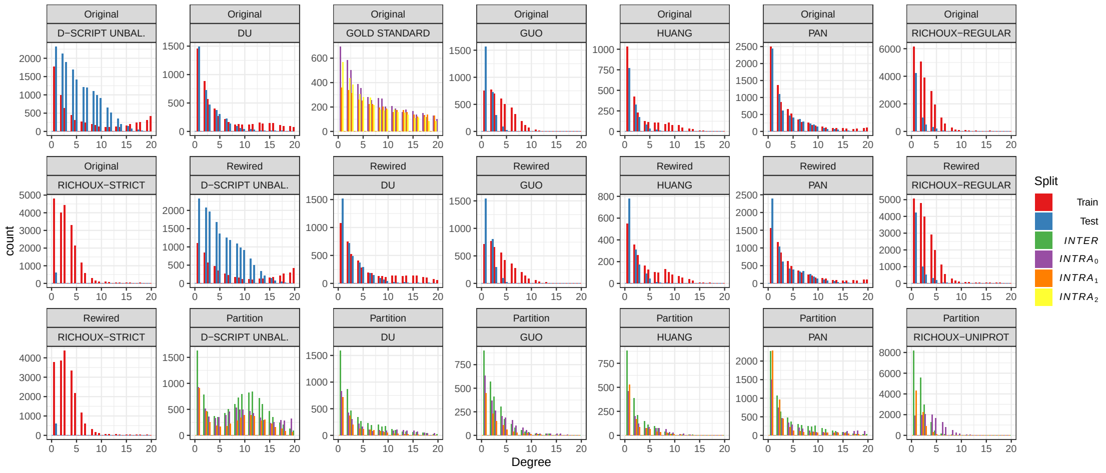

Figure S4: Comparison of node degrees between training and test sets (original/rewired) and block $INTRA_0$ vs. $INTRA_1$ vs. $INTER$. The distributions all follow approximately the same trends.

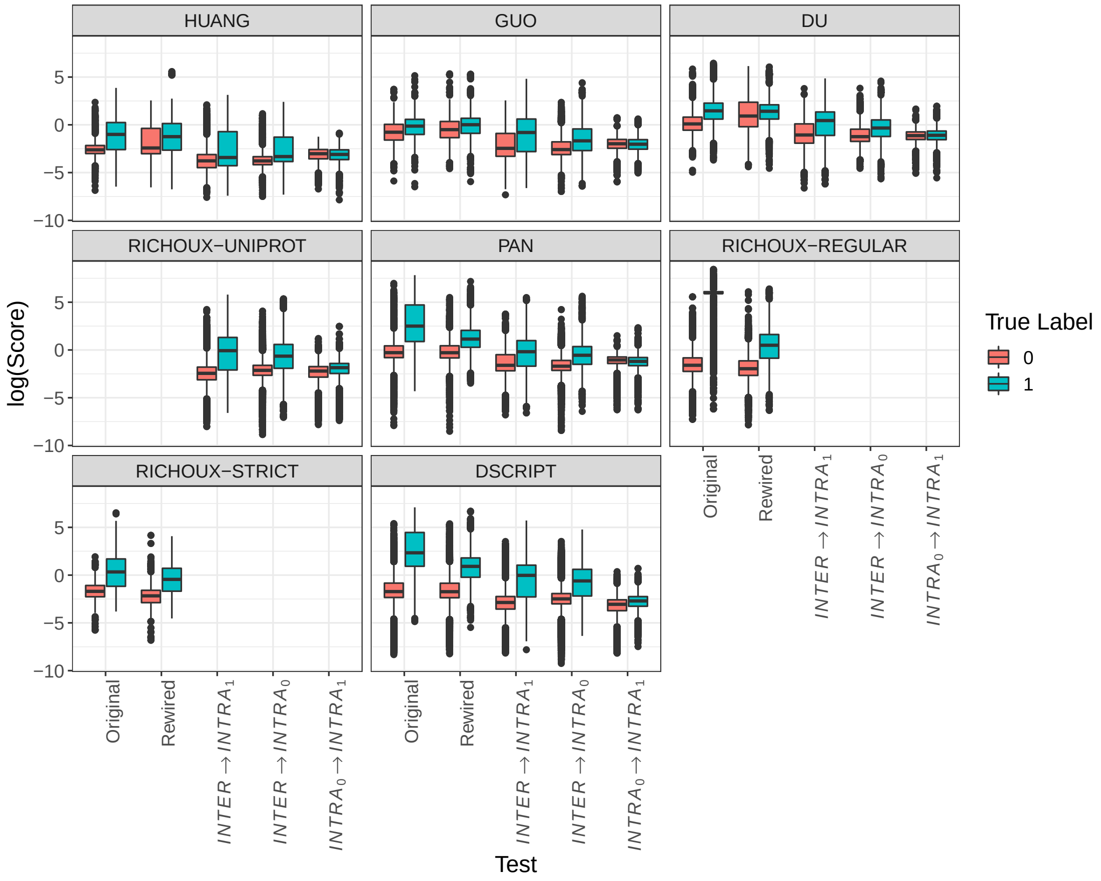

Figure S5: Distribution of SPRINT scores. For almost all original datasets, scores assigned to positive PPIs are significantly higher than scores assigned to negative examples. This difference drops strongly for the rewired test sets and the $INTER \to INTRA_0$/ $INTRA_1$ prediction (the y-axis is logged). For $INTRA_0 \to INTRA_1$, no difference between scores for positive and negative samples can be seen, resulting in random performance measures.

Loc./ Glob. Cons.	D-SCRIPT	SVM-MDS	RF-node2vec	RF-MDS	SVM-PCA	Richoux-LSTM	SPRINT	SVM-node2vec	Deep FE	RF-PCA	PIPR	Richoux-FC	Topsy-Turvy
0.50	0.50	0.50	0.51	0.51	0.51	0.52	0.52	0.52	0.52	0.52	0.52	0.52	0.56

Table S5: Accuracies of all methods evaluated on the Gold Standard test set. Richoux-LSTM, DeepFE, Richoux-FC, and PIPR were trained with the training set and validated on the validation set. The other methods do not have tunable hyperparameters and hence were trained on the collapsed training and validation set.

Loc./ Glob. Cons.	Deep FE	SVM-MDS	RF-node2vec	RF-MDS	SVM-PCA	SPRINT	SVM-node2vec	RF-PCA	Richoux-LSTM	PIPR	Richoux-FC	Topsy-Turvy	D-SCRIPT
0.50	0.50	0.50	0.51	0.51	0.51	0.52	0.52	0.52	0.52	0.52	0.53	0.53	0.55

Table S6: Accuracies of all methods evaluated on the Gold Standard test set after early stopping.

Table S7: Epochs in which early stopping was triggered.

Model	Dataset	Original	Rewired	Partition
DeepFE	D-SCRIPT UNBAL.	15	16	$INTER \rightarrow INTRA_0$: 7; $INTER \rightarrow INTRA_1$: 9; $INTRA_0 \rightarrow INTRA_1$: 11
DeepFE	DU	1	1	$INTER \rightarrow INTRA_0$: 1; $INTER \rightarrow INTRA_1$: 1; INTRA0->INTRA1: 2
DeepFE	GUO	2	1	$INTER \rightarrow INTRA_0$: 2; $INTER \rightarrow INTRA_1$: 3; INTRA0->INTRA1: 3
DeepFE	HUANG	5	12	$INTER \rightarrow INTRA_0$: 1; $INTER \rightarrow INTRA_1$: 2; INTRA0->INTRA1: 3
DeepFE	PAN	1	7	$INTER \rightarrow INTRA_0$: 2; $INTER \rightarrow INTRA_1$: 2; INTRA0->INTRA1: 1
DeepFE	RICHOUX			$INTER \rightarrow INTRA_0$: 2; $INTER \rightarrow INTRA_1$: 1; INTRA0->INTRA1: 1
DeepFE	RICHOUX-REGULAR	1	1	
DeepFE	RICHOUX-STRICT	1	1	
PIPR	D-SCRIPT UNBAL.	10	1	$INTER \rightarrow INTRA_0$: 1; $INTER \rightarrow INTRA_1$: 2; INTRA0->INTRA1: 1
PIPR	DU	9	4	$INTER \rightarrow INTRA_0$: 6; $INTER \rightarrow INTRA_1$: 16; INTRA0->INTRA1: 10
PIPR	GUO	1	1	$INTER \rightarrow INTRA_0$: 12; $INTER \rightarrow INTRA_1$: 15; INTRA0->INTRA1: 6
PIPR	HUANG	28	1	$INTER \rightarrow INTRA_0$: 6; $INTER \rightarrow INTRA_1$: 1; INTRA0->INTRA1: 14
PIPR	PAN	3	8	$INTER \rightarrow INTRA_0$: 22; $INTER \rightarrow INTRA_1$: 9; INTRA0->INTRA1: 7
PIPR	RICHOUX			$INTER \rightarrow INTRA_0$: 12; $INTER \rightarrow INTRA_1$: 8; INTRA0->INTRA1: 6
PIPR	RICHOUX-REGULAR	13	4	

Table S7: Epochs in which early stopping was triggered.

PIPR	RICHOUX-STRICT	6	7	
Richoux FC	D-SCRIPT UNBAL.	1	1	*INTER* $\rightarrow$ *INTRA*$_0$: 1; *INTER* $\rightarrow$ *INTRA*$_1$: 2; INTRA0->INTRA1: 2
Richoux FC	DU	1	1	*INTER* $\rightarrow$ *INTRA*$_0$: 1; *INTER* $\rightarrow$ *INTRA*$_1$: 3; INTRA0->INTRA1: 1
Richoux FC	GUO	1	1	*INTER* $\rightarrow$ *INTRA*$_0$: 1; *INTER* $\rightarrow$ *INTRA*$_1$: 25; INTRA0->INTRA1: 1
Richoux FC	HUANG	1	1	*INTER* $\rightarrow$ *INTRA*$_0$: 1; *INTER* $\rightarrow$ *INTRA*$_1$: 1; INTRA0->INTRA1: 3
Richoux FC	PAN	16	1	*INTER* $\rightarrow$ *INTRA*$_0$: 1; *INTER* $\rightarrow$ *INTRA*$_1$: 25; INTRA0->INTRA1: 25
Richoux FC	RICHOUX			*INTER* $\rightarrow$ *INTRA*$_0$: 7; *INTER* $\rightarrow$ *INTRA*$_1$: 5; INTRA0->INTRA1: 4
Richoux FC	RICHOUX-REGULAR	10	11	
Richoux FC	RICHOUX-STRICT	1	1	
Richoux LSTM	D-SCRIPT UNBAL.	1	1	*INTER* $\rightarrow$ *INTRA*$_0$: 1; *INTER* $\rightarrow$ *INTRA*$_1$: 1; INTRA0->INTRA1: 1
Richoux LSTM	DU	1	1	*INTER* $\rightarrow$ *INTRA*$_0$: 1; *INTER* $\rightarrow$ *INTRA*$_1$: 1; INTRA0->INTRA1: 1
Richoux LSTM	GUO	1	1	*INTER* $\rightarrow$ *INTRA*$_0$: 1; *INTER* $\rightarrow$ *INTRA*$_1$: 1; INTRA0->INTRA1: 1
Richoux LSTM	HUANG	1	1	*INTER* $\rightarrow$ *INTRA*$_0$: 1; *INTER* $\rightarrow$ *INTRA*$_1$: 1; INTRA0->INTRA1: 1
Richoux LSTM	PAN	1	24	*INTER* $\rightarrow$ *INTRA*$_0$: 1; *INTER* $\rightarrow$ *INTRA*$_1$: 1; INTRA0->INTRA1: 1
Richoux LSTM	RICHOUX			*INTER* $\rightarrow$ *INTRA*$_0$: 1; *INTER* $\rightarrow$ *INTRA*$_1$: 8; INTRA0->INTRA1: 13
Richoux LSTM	RICHOUX-REGULAR	17	16	
Richoux LSTM	RICHOUX-STRICT	1	19	
D-SCRIPT	D-SCRIPT UNBAL.	7	2	*INTER* $\rightarrow$ *INTRA*$_0$: 10; *INTER* $\rightarrow$ *INTRA*$_1$: 4; INTRA0->INTRA1: 1
D-SCRIPT	DU	9	9	*INTER* $\rightarrow$ *INTRA*$_0$: 10; *INTER* $\rightarrow$ *INTRA*$_1$: 10; INTRA0->INTRA1: 5
D-SCRIPT	GUO	10	9	*INTER* $\rightarrow$ *INTRA*$_0$: 6; *INTER* $\rightarrow$ *INTRA*$_1$: 3; INTRA0->INTRA1: 2
D-SCRIPT	HUANG	5	8	*INTER* $\rightarrow$ *INTRA*$_0$: 8; *INTER* $\rightarrow$ *INTRA*$_1$: 9; INTRA0->INTRA1: 8
D-SCRIPT	PAN	9	6	*INTER* $\rightarrow$ *INTRA*$_0$: 8; *INTER* $\rightarrow$ *INTRA*$_1$: 2; INTRA0->INTRA1: 1
D-SCRIPT	RICHOUX			*INTER* $\rightarrow$ *INTRA*$_0$: 6; *INTER* $\rightarrow$ *INTRA*$_1$: 1; INTRA0->INTRA1: 3
D-SCRIPT	RICHOUX-REGULAR	1	1	
D-SCRIPT	RICHOUX-STRICT	1	1	
Topsy-Turvy	D-SCRIPT UNBAL.	4	3	*INTER* $\rightarrow$ *INTRA*$_0$: 5; *INTER* $\rightarrow$ *INTRA*$_1$: 7; INTRA0->INTRA1: 1
Topsy-Turvy	DU	3	10	*INTER* $\rightarrow$ *INTRA*$_0$: 1; *INTER* $\rightarrow$ *INTRA*$_1$: 4; INTRA0->INTRA1: 5

Table S7: Epochs in which early stopping was triggered.

Topsy-Turvy	GUO	7	9	$INTER \rightarrow INTRA_0$: 8; $INTER \rightarrow INTRA_1$: 3; INTRA0->INTRA1: 3
Topsy-Turvy	HUANG	7	4	$INTER \rightarrow INTRA_0$: 10; $INTER \rightarrow INTRA_1$: 1; INTRA0->INTRA1: 9
Topsy-Turvy	PAN	2	7	$INTER \rightarrow INTRA_0$: 7; $INTER \rightarrow INTRA_1$: 9; INTRA0->INTRA1: 1
Topsy-Turvy	RICHOUX			$INTER \rightarrow INTRA_0$: 9; $INTER \rightarrow INTRA_1$: 2; INTRA0->INTRA1: 10
Topsy-Turvy	RICHOUX-REGULAR	1	1	
Topsy-Turvy	RICHOUX-STRICT	5	1	

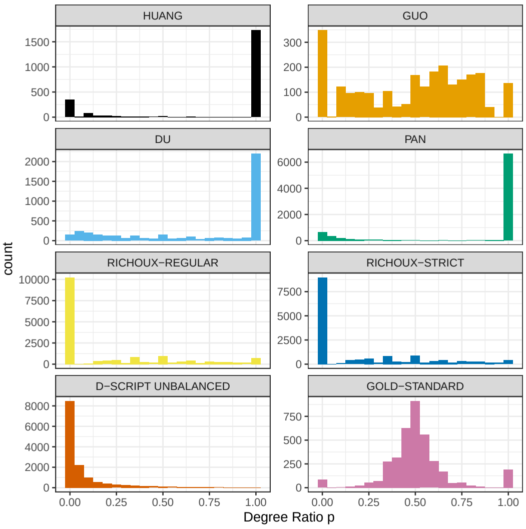

(a)

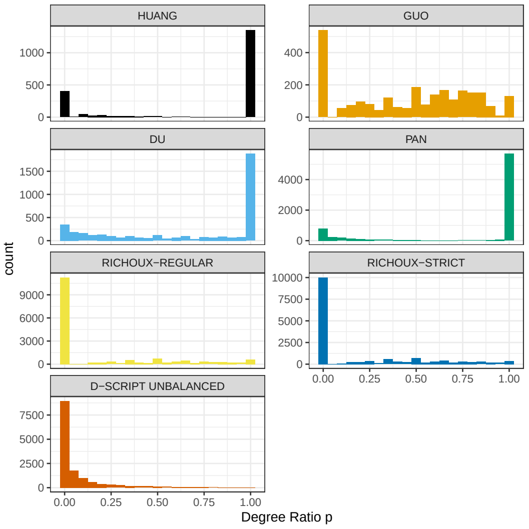

(b)

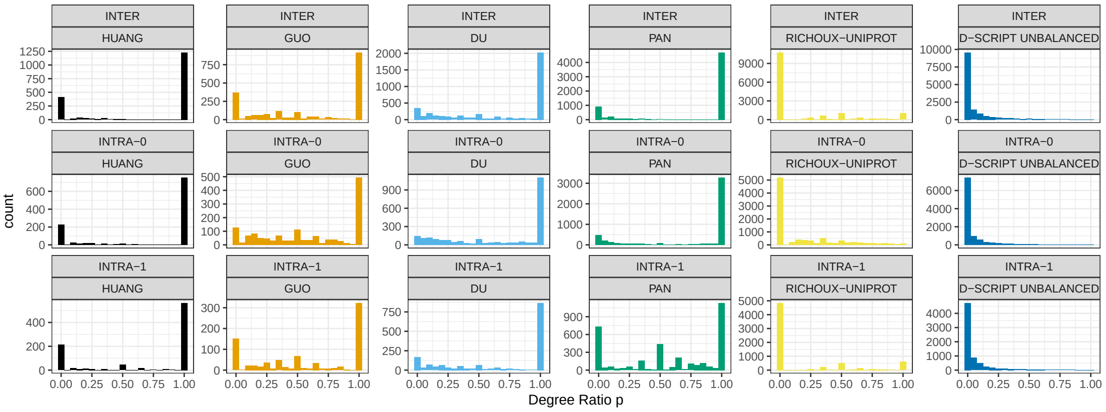

(c)

Figure S6: Degree ratios for the training datasets for the **(a)** original, **(b)** rewired, and **(c)** partition datasets. Most of the proteins in HUANG and PAN have either exclusively positive or negative interactions. DU has many proteins that only have positive interactions, the RICHOUX datasets have many proteins with only negative annotations.

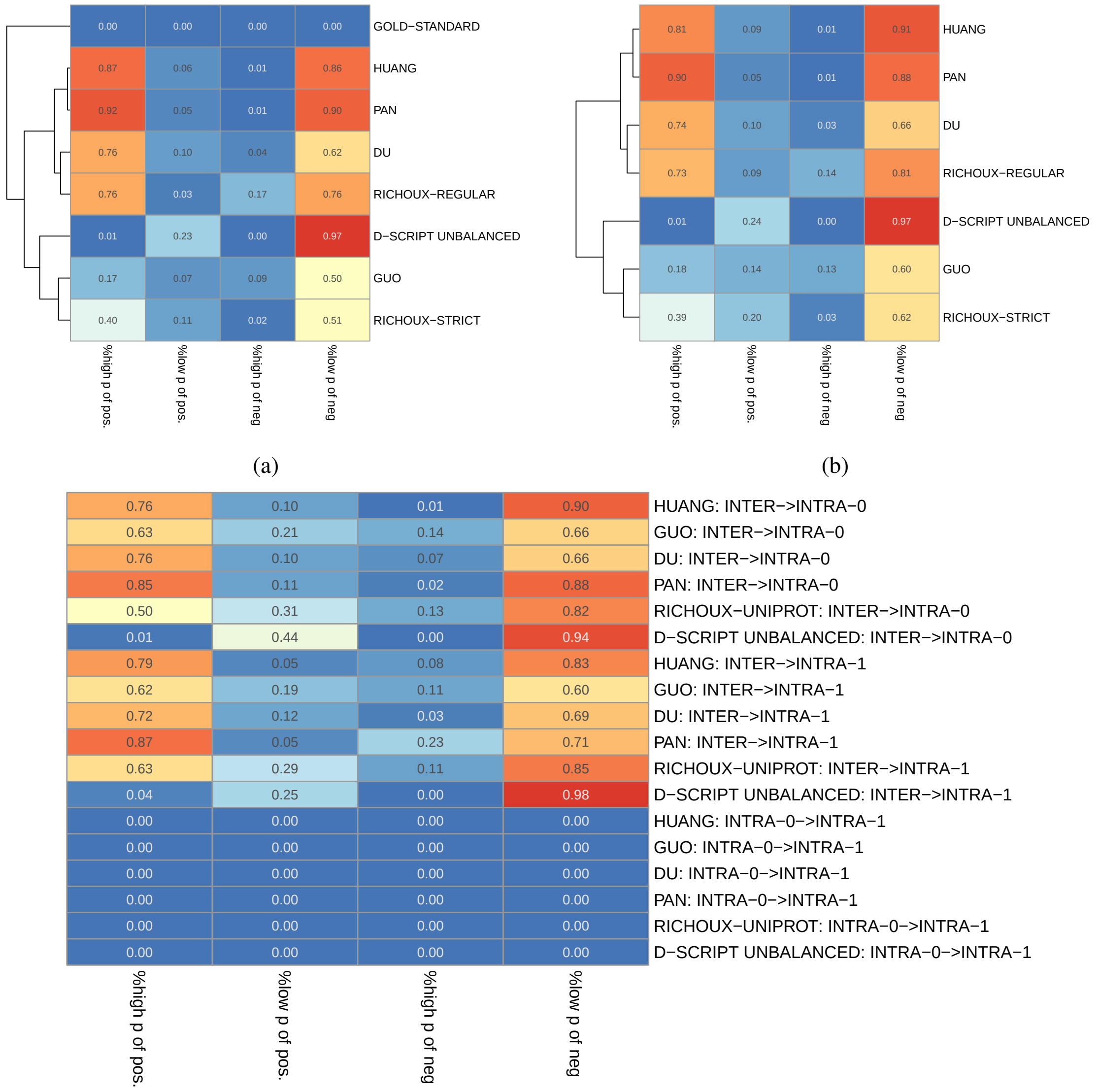

(c)

Figure S7: Proportions of training proteins with a high (≥ 0.9) or low (≤ 0.1) degree ratio in the positive and negative parts of the test sets displayed for the **(a)** original, **(b)** rewired, and **(c)** partition datasets. Consequently, especially HUANG, and PAN should be very easy to predict. RICHOUX-REGULAR and DU should be a bit harder and GUO and RICHOUX-STRICT the hardest. This trend is visible in Figure 2a and 3a.

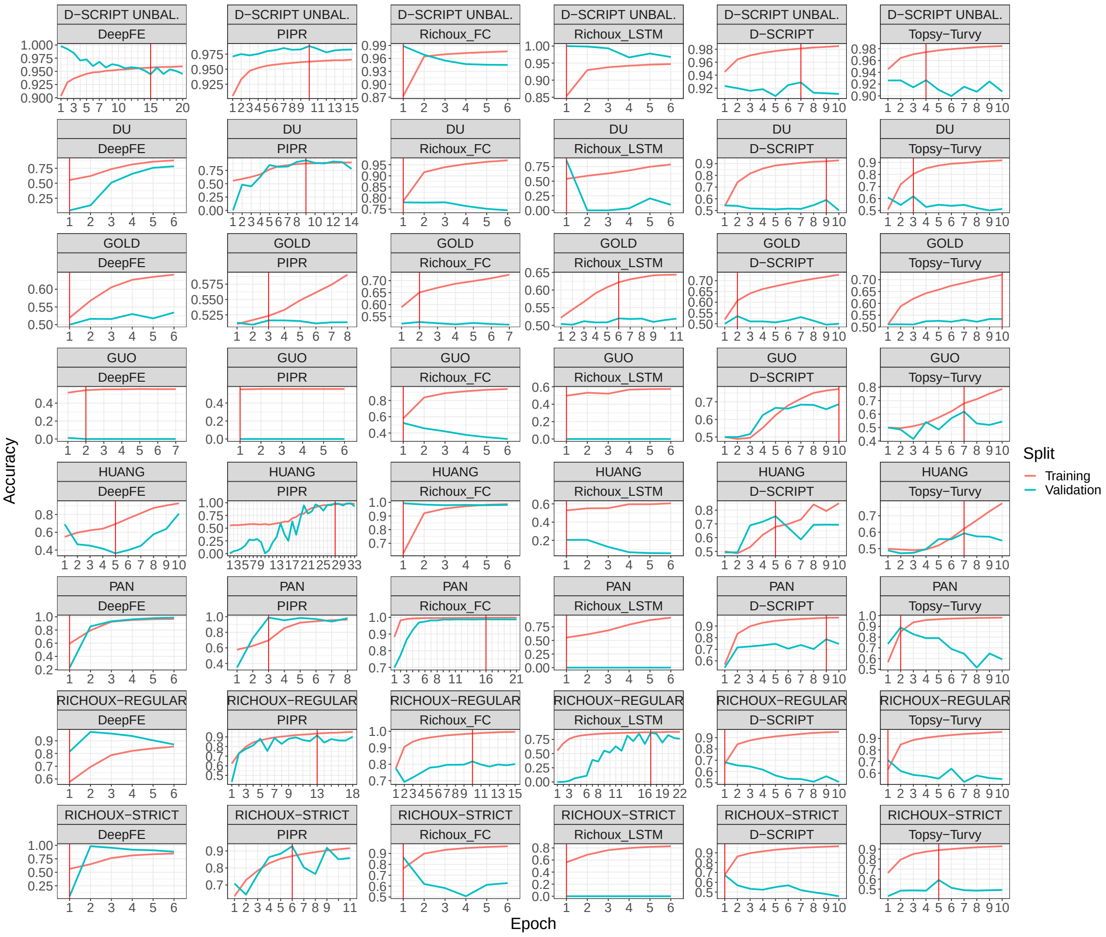

Figure S8: Training and validation accuracy over all epochs until early stopping for all deep learning methods on the original datasets. The red line indicates which model was used to evaluate the test set in the early stopping setting.

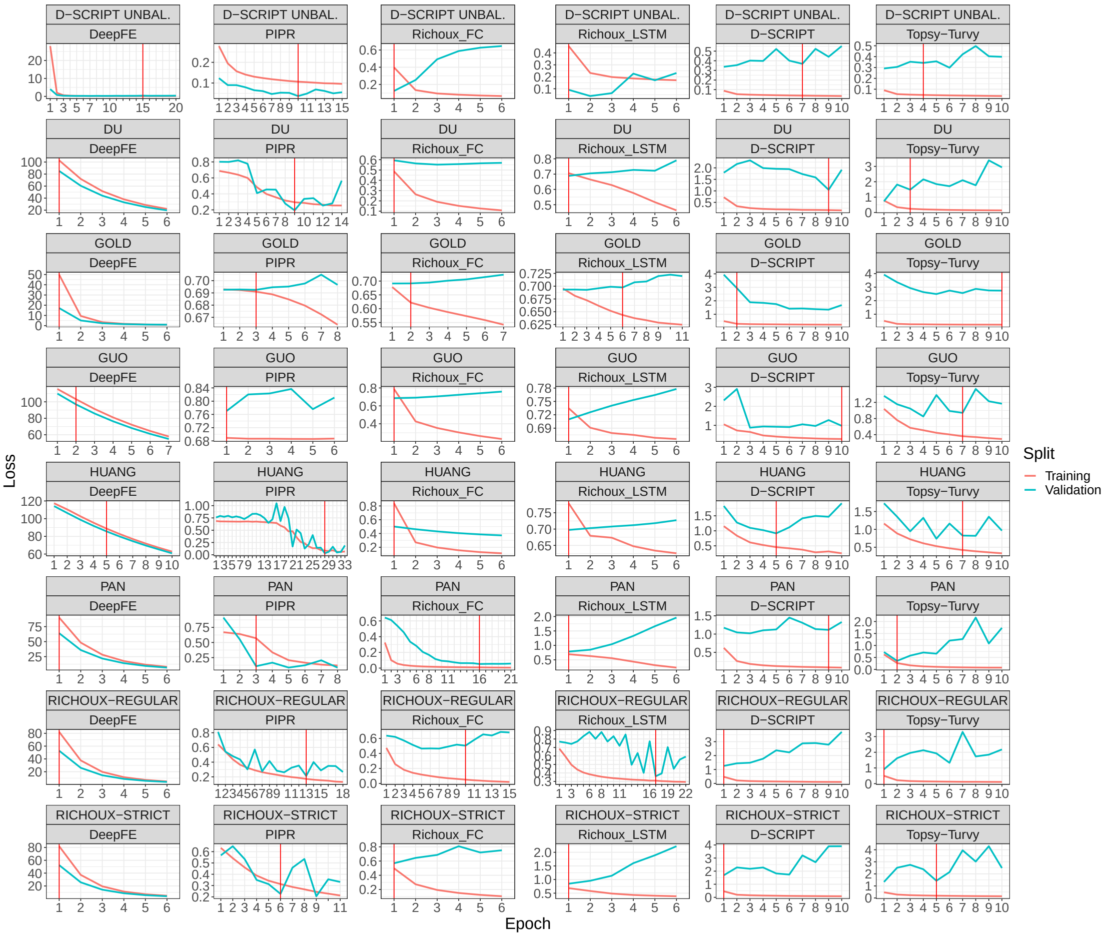

Figure S9: Training and validation loss over all epochs until early stopping for all deep learning methods on the original datasets. The red line indicates which model was used to evaluate the test set in the early stopping setting.

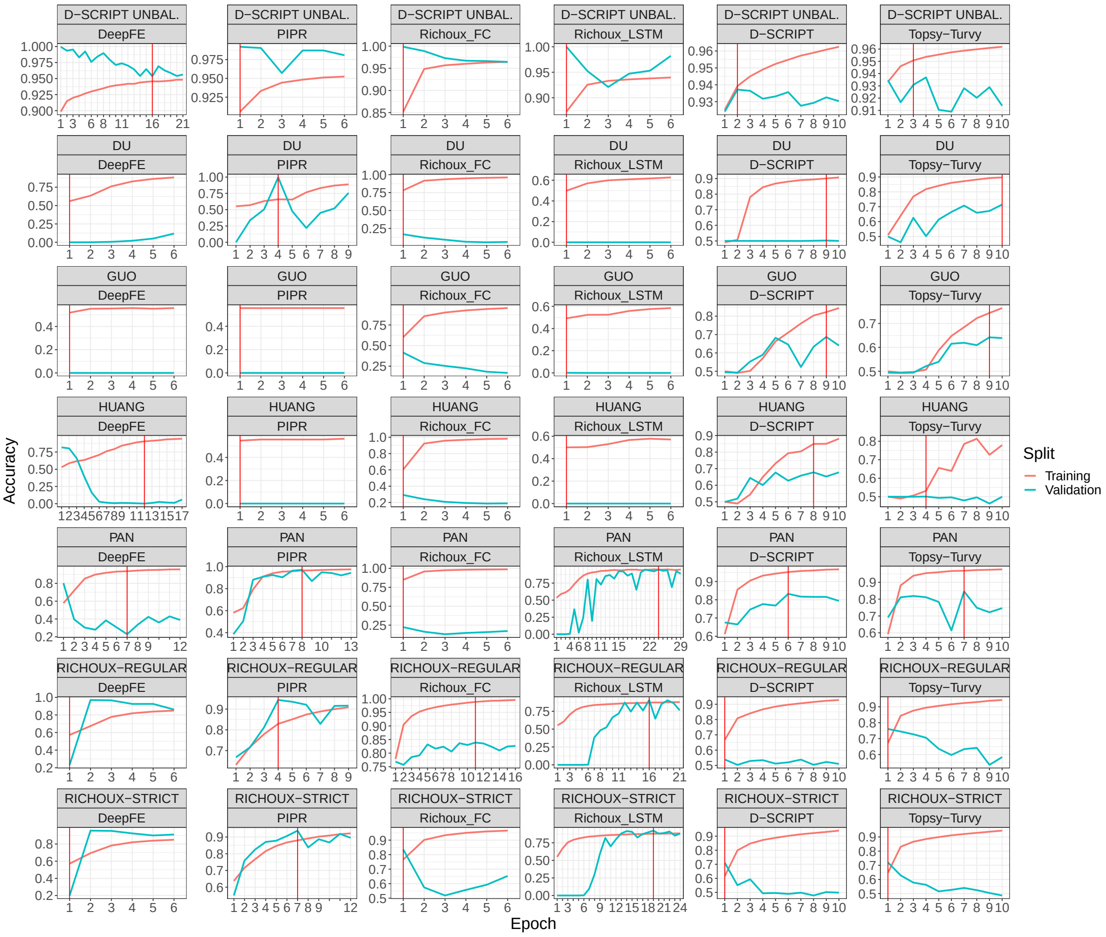

Figure S10: Training and validation accuracy over all epochs until early stopping for all deep learning methods on the rewired datasets. The red line indicates which model was used to evaluate the test set in the early stopping setting.

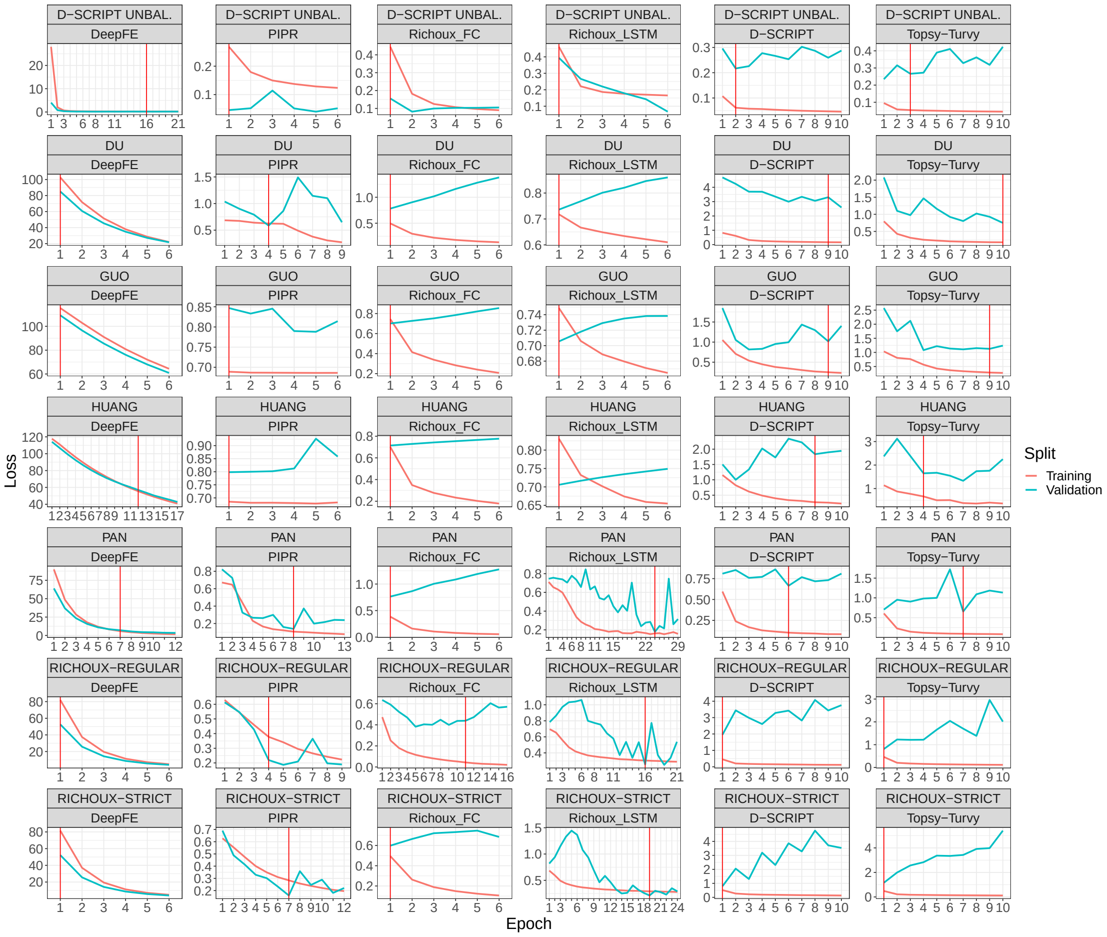

Figure S11: Training and validation loss over all epochs until early stopping for all deep learning methods on the rewired datasets. The red line indicates which model was used to evaluate the test set in the early stopping setting.

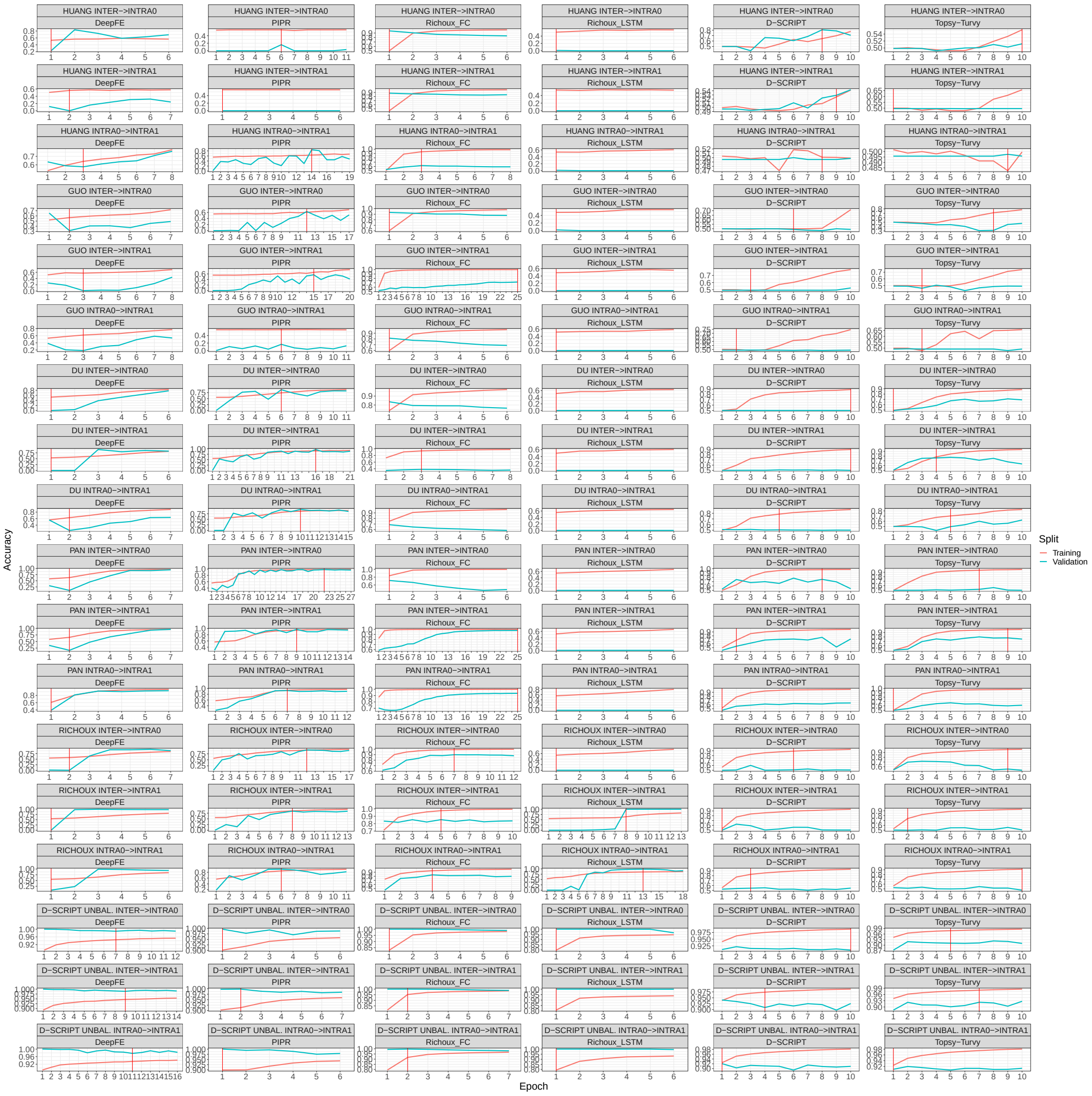

Figure S12: Training and validation accuracy over all epochs until early stopping for all deep learning methods on the partition datasets. The red line indicates which model was used to evaluate the test set in the early stopping setting.

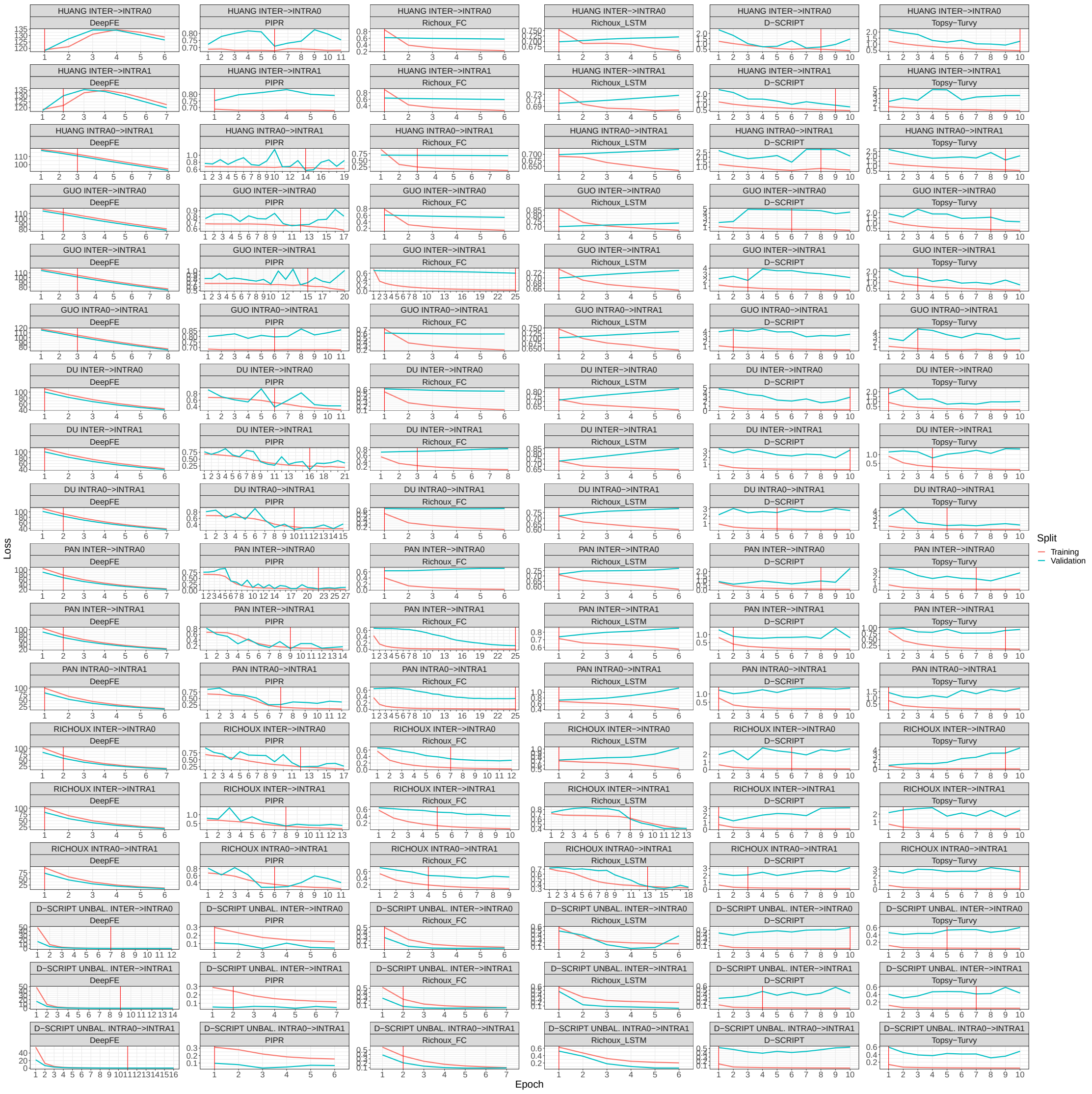

Figure S13: Training and validation loss over all epochs until early stopping for all deep learning methods on the partition datasets. The red line indicates which model was used to evaluate the test set in the early stopping setting.

Test	Dataset	SPRINT (AUPR)	Richoux-FC	Richoux-LSTM	DeepFE	PIPR	D-SCRIPT	Topsy Turvy	RF-PCA	SVM-PCA	RF-MDS	SVM-MDS	RF-node2vec	SVM-node2vec	Harmonic Function	Global and Local Consistency
Original	GOLD STANDARD (222,452/95,018)	0.51	0.52	0.52	0.52	0.52	0.50	0.56	0.52	0.51	0.51	0.50	0.51	0.51	NA	0.50
Original	HUANG (5,316/3,418)	0.75	0.97	0.94	0.97	0.95	0.70	0.56	0.97	0.65	0.97	0.95	0.95	0.82	0.92	0.88
Original	GUO (8,966/6,309)	0.63	0.70	0.91	0.88	0.96	0.69	0.54	0.71	0.52	0.72	0.62	0.70	0.60	0.81	0.74
Original	DU (27,514/19,566)	0.73	0.91	0.88	0.91	0.89	0.50	0.51	0.88	0.45	0.88	0.69	0.88	0.65	0.87	0.84
Original	PAN (50,414/32,405)	0.89	0.99	0.88	0.98	0.98	0.75	0.59	0.97	0.40	0.98	0.76	0.97	0.47	0.93	0.89
Original	RICHOUX−REGULAR (67,404/59,842)	0.99	0.88	0.85	0.88	0.91	0.51	0.55	0.92	0.50	0.92	0.62	0.91	0.48	0.80	0.78
Original	RICHOUX−STRICT (68,144/60,555)	0.81	0.74	0.73	0.69	0.72	0.47	0.50	0.73	0.47	0.71	0.58	0.72	0.47	0.73	0.67
Original	D−SCRIPT UNBALANCED (379,247/341,193)	0.78	0.88	0.83	0.87	0.86	0.52	0.55	0.80	0.52	0.78	0.62	0.78	0.56	NA	0.50
Rewired	HUANG (5,356/3,447)	0.56	0.89	0.90	0.88	0.91	0.75	0.50	0.85	0.46	0.88	0.83	0.86	0.63	0.91	0.91
Rewired	GUO (8,966/6,350)	0.58	0.72	0.76	0.75	0.85	0.59	0.51	0.66	0.52	0.66	0.63	0.66	0.58	0.75	0.64
Rewired	DU (27,458/19,510)	0.34	0.83	0.87	0.85	0.88	0.51	0.54	0.76	0.45	0.76	0.64	0.77	0.62	0.87	0.85
Rewired	PAN (50,392/31,800)	0.81	0.94	0.94	0.93	0.97	0.64	0.58	0.88	0.42	0.84	0.64	0.89	0.47	0.92	0.90
Rewired	RICHOUX−REGULAR (67,592/59,925)	0.84	0.84	0.83	0.84	0.85	0.52	0.52	0.84	0.50	0.87	0.61	0.82	0.46	0.81	0.75
Rewired	RICHOUX−STRICT (68,268/60,656)	0.73	0.70	0.64	0.64	0.65	0.49	0.50	0.71	0.46	0.71	0.54	0.72	0.49	0.71	0.68
Rewired	D−SCRIPT UNBALANCED (379,764/341,688)	0.50	0.80	0.82	0.80	0.83	0.51	0.53	0.69	0.53	0.68	0.59	0.67	0.55	NA	0.50
Inter−>Intra−1	HUANG (2,850/1,861)	0.62	0.93	0.80	0.93	0.87	0.49	0.49	0.89	0.71	0.92	0.87	0.89	0.77	0.90	0.90
Inter−>Intra−1	GUO (4,604/3,169)	0.68	0.87	0.81	0.89	0.82	0.50	0.55	0.89	0.50	0.90	0.84	0.90	0.80	0.80	0.75
Inter−>Intra−1	DU (15,202/10,922)	0.72	0.90	0.86	0.89	0.87	0.50	0.56	0.87	0.46	0.87	0.73	0.87	0.70	0.84	0.78
Inter−>Intra−1	PAN (22,596/15,053)	0.72	0.85	0.81	0.84	0.83	0.50	0.76	0.83	0.38	0.84	0.77	0.83	0.44	0.82	0.81
Inter−>Intra−1	RICHOUX−UNIPROT (28,866/24,579)	0.79	0.81	0.79	0.80	0.81	0.52	0.68	0.78	0.51	0.81	0.66	0.76	0.53	0.77	0.71
Inter−>Intra−1	D−SCRIPT UNBALANCED (33,348/164,973)	0.49	0.84	0.84	0.85	0.83	0.52	0.59	0.72	0.61	0.77	0.64	0.73	0.56	NA	0.50
Inter−>Intra−0	HUANG (2,850/1,861)	0.69	0.97	0.50	0.95	0.80	0.50	0.50	0.95	0.74	0.95	0.85	0.94	0.75	0.90	0.83
Inter−>Intra−0	GUO (4,604/3,169)	0.68	0.85	0.52	0.85	0.80	0.51	0.48	0.87	0.52	0.87	0.78	0.87	0.78	0.78	0.75
Inter−>Intra−0	DU (15,202/10,922)	0.70	0.88	0.85	0.87	0.86	0.71	0.55	0.87	0.44	0.86	0.71	0.87	0.67	0.87	0.85
Inter−>Intra−0	PAN (22,596/15,053)	0.76	0.96	0.91	0.96	0.93	0.71	0.63	0.96	0.40	0.96	0.76	0.94	0.53	0.90	0.86
Inter−>Intra−0	RICHOUX−UNIPROT (28,866/24,579)	0.79	0.78	0.73	0.77	0.76	0.50	0.57	0.77	0.48	0.78	0.60	0.75	0.48	0.73	0.67
Inter−>Intra−0	D−SCRIPT UNBALANCED (33,348/164,973)	0.37	0.78	0.71	0.77	0.75	0.61	0.51	0.61	0.54	0.72	0.61	0.62	0.56	NA	0.50
Intra−0−>Intra−1	HUANG (2,410/1,346)	0.48	0.51	0.48	0.52	0.52	0.50	0.52	0.57	0.56	0.50	0.50	0.52	0.57	0.50	0.50
Intra−0−>Intra−1	GUO (4,640/3,193)	0.52	0.51	0.51	0.55	0.58	0.50	0.55	0.51	0.41	0.49	0.51	0.52	0.56	0.50	0.50
Intra−0−>Intra−1	DU (14,468/9,862)	0.54	0.52	0.50	0.54	0.56	0.50	0.50	0.50	0.46	0.50	0.50	0.50	0.54	0.50	0.50
Intra−0−>Intra−1	PAN (31,212/18,991)	0.48	0.48	0.51	0.50	0.50	0.50	0.53	0.53	0.42	0.50	0.50	0.52	0.44	0.50	0.50
Intra−0−>Intra−1	RICHOUX−UNIPROT (39,634/33,076)	0.60	0.52	0.52	0.53	0.56	0.49	0.48	0.50	0.51	0.50	0.52	0.50	0.54	0.50	0.50
Intra−0−>Intra−1	D−SCRIPT UNBALANCED (27,148/134,293)	0.17	0.50	0.52	0.51	0.52	0.53	0.51	0.50	0.60	0.50	0.50	0.50	0.56	NA	0.50

Figure S14: Balanced accuracies (AUPRs for SPRINT) for all methods and datasets.

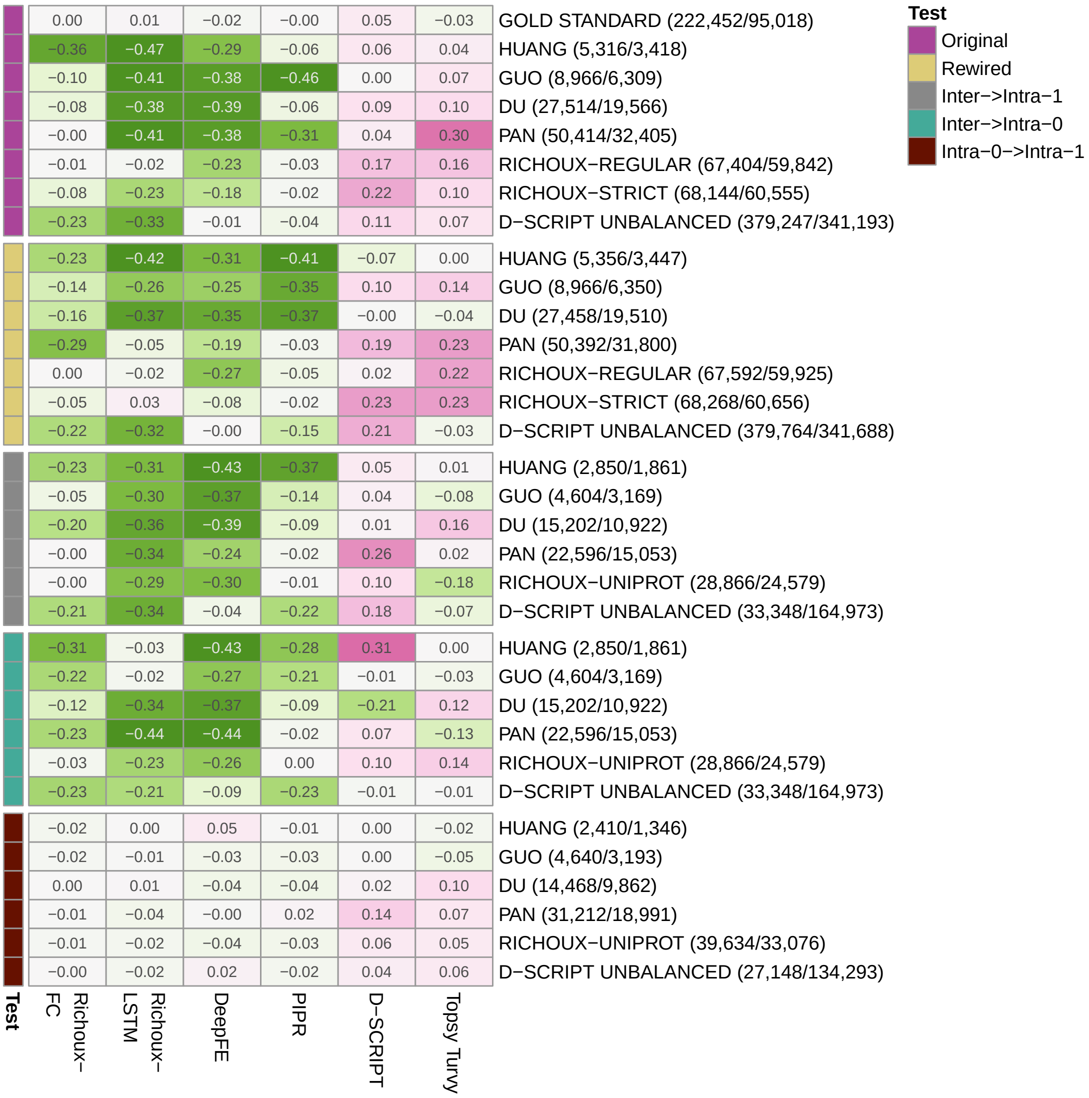

Figure S15: Difference between the balanced accuracies from the early stopping setting and the balanced accuracies obtained without early stopping. Only D-SCRIPT and Topsy-Turvy seem to profit from early stopping.

Test	Dataset	SPRINT (AUPR)	Richoux−FC	Richoux−LSTM	DeepFE	PIPR	D−SCRIPT	Topsy Turvy	RF−PCA	SVM−PCA	RF−MDS	SVM−MDS	RF−node2vec	SVM−node2vec	Harmonic Function	Global and Local Consistency
Original	GOLD STANDARD (222,452/95,018)	0.51	0.53	0.51	0.52	0.52	0.51	0.65	0.54	0.51	0.52	0.50	0.53	0.51	NA	0.50
Original	HUANG (5,316/3,418)	0.75	0.97	0.98	0.99	0.97	0.96	0.89	0.98	0.69	0.97	0.96	0.96	0.79	0.96	0.97
Original	GUO (8,966/6,309)	0.63	0.64	0.94	0.91	0.97	0.75	0.64	0.70	0.51	0.72	0.62	0.71	0.61	0.79	0.71
Original	DU (27,514/19,566)	0.73	0.90	0.90	0.90	0.91	0.91	0.89	0.89	0.47	0.89	0.66	0.89	0.67	0.90	0.93
Original	PAN (50,414/32,405)	0.89	0.99	0.86	0.99	0.99	0.91	0.87	0.98	0.42	0.98	0.78	0.98	0.47	0.97	0.98
Original	RICHOUX−REGULAR (67,404/59,842)	0.99	0.82	0.84	0.85	0.87	0.80	0.89	0.89	0.50	0.88	0.64	0.87	0.48	0.76	0.72
Original	RICHOUX−STRICT (68,144/60,555)	0.81	0.84	0.81	0.79	0.85	0.27	0.57	0.85	0.48	0.85	0.58	0.83	0.46	0.71	0.63
Original	D−SCRIPT UNBALANCED (379,247/341,193)	0.78	0.81	0.85	0.86	0.86	0.73	0.47	0.94	0.10	0.91	0.16	0.92	0.11	NA	NA
Rewired	HUANG (5,356/3,447)	0.56	0.91	0.86	0.85	0.98	0.71	0.00	0.91	0.48	0.88	0.85	0.92	0.61	0.96	0.97
Rewired	GUO (8,966/6,350)	0.58	0.75	0.83	0.76	0.84	0.84	0.60	0.68	0.51	0.69	0.64	0.68	0.57	0.74	0.61
Rewired	DU (27,458/19,510)	0.34	0.84	0.92	0.86	0.91	0.62	0.82	0.74	0.46	0.73	0.63	0.74	0.62	0.91	0.93
Rewired	PAN (50,392/31,800)	0.81	0.94	0.97	0.93	0.99	0.89	0.95	0.87	0.43	0.80	0.66	0.91	0.47	0.97	0.97
Rewired	RICHOUX−REGULAR (67,592/59,925)	0.84	0.81	0.84	0.83	0.84	0.89	0.87	0.85	0.50	0.87	0.67	0.84	0.46	0.74	0.67
Rewired	RICHOUX−STRICT (68,268/60,656)	0.73	0.83	0.73	0.73	0.89	0.00	0.57	0.83	0.46	0.86	0.54	0.83	0.49	0.69	0.64
Rewired	D−SCRIPT UNBALANCED (379,764/341,688)	0.50	0.73	0.78	0.77	0.79	0.86	0.80	0.83	0.10	0.86	0.15	0.86	0.10	NA	NA
Inter−>Intra−1	HUANG (2,850/1,861)	0.62	0.88	0.91	0.90	0.89	0.31	0.31	0.90	0.87	0.90	0.89	0.89	0.81	0.89	0.91
Inter−>Intra−1	GUO (4,604/3,169)	0.68	0.86	0.91	0.91	0.84	0.22	0.83	0.89	0.50	0.89	0.87	0.91	0.86	0.90	0.91
Inter−>Intra−1	DU (15,202/10,922)	0.72	0.90	0.94	0.90	0.87	0.38	0.83	0.90	0.48	0.90	0.79	0.90	0.78	0.91	0.93
Inter−>Intra−1	PAN (22,596/15,053)	0.72	0.76	0.76	0.77	0.77	0.78	0.78	0.77	0.41	0.76	0.74	0.76	0.44	0.78	0.78
Inter−>Intra−1	RICHOUX−UNIPROT (28,866/24,579)	0.79	0.84	0.83	0.82	0.88	0.93	0.73	0.89	0.51	0.88	0.64	0.87	0.53	0.73	0.65
Inter−>Intra−1	D−SCRIPT UNBALANCED (33,348/164,973)	0.49	0.85	0.83	0.84	0.81	0.86	0.47	0.91	0.12	0.92	0.17	0.89	0.11	NA	NA
Inter−>Intra−0	HUANG (2,850/1,861)	0.69	0.96	0.50	0.95	0.73	0.00	0.00	0.94	0.77	0.96	0.86	0.94	0.76	0.95	0.98
Inter−>Intra−0	GUO (4,604/3,169)	0.68	0.84	0.51	0.88	0.82	0.64	0.47	0.88	0.52	0.86	0.78	0.87	0.80	0.85	0.87
Inter−>Intra−0	DU (15,202/10,922)	0.70	0.86	0.84	0.90	0.85	0.88	0.76	0.86	0.45	0.85	0.77	0.85	0.68	0.91	0.93
Inter−>Intra−0	PAN (22,596/15,053)	0.76	0.96	0.94	0.96	0.93	0.98	0.95	0.95	0.41	0.96	0.73	0.94	0.53	0.95	0.96
Inter−>Intra−0	RICHOUX−UNIPROT (28,866/24,579)	0.79	0.79	0.82	0.77	0.80	0.00	0.94	0.80	0.48	0.87	0.60	0.74	0.48	0.66	0.61
Inter−>Intra−0	D−SCRIPT UNBALANCED (33,348/164,973)	0.37	0.63	0.53	0.44	0.63	0.86	0.75	0.83	0.11	0.65	0.16	0.86	0.12	NA	NA
Intra−0−>Intra−1	HUANG (2,410/1,346)	0.48	0.50	0.48	0.51	0.52	0.00	0.76	0.55	0.58	0.50	0.50	0.51	0.56	0.50	0.50
Intra−0−>Intra−1	GUO (4,640/3,193)	0.52	0.51	0.54	0.54	0.57	0.44	0.76	0.51	0.41	0.50	0.51	0.51	0.57	0.50	0.50
Intra−0−>Intra−1	DU (14,468/9,862)	0.54	0.51	0.50	0.53	0.56	0.50	0.47	0.50	0.47	0.50	0.50	0.50	0.53	0.50	0.50
Intra−0−>Intra−1	PAN (31,212/18,991)	0.48	0.48	0.49	0.50	0.50	0.52	0.62	0.51	0.41	0.50	0.50	0.51	0.41	0.50	0.50
Intra−0−>Intra−1	RICHOUX−UNIPROT (39,634/33,076)	0.60	0.70	0.56	0.62	0.74	0.48	0.38	0.68	0.51	0.76	0.52	0.58	0.53	0.50	0.50
Intra−0−>Intra−1	D−SCRIPT UNBALANCED (27,148/134,293)	0.17	0.30	0.19	0.22	0.26	0.90	0.57	NA	0.13	NA	0.09	0.25	0.11	NA	0.09

Test: Original; Rewired; Inter−>Intra−1; Inter−>Intra−0; Intra−0−>Intra−1

Figure S16: Precision (AUPRs for SPRINT) for all methods and datasets.

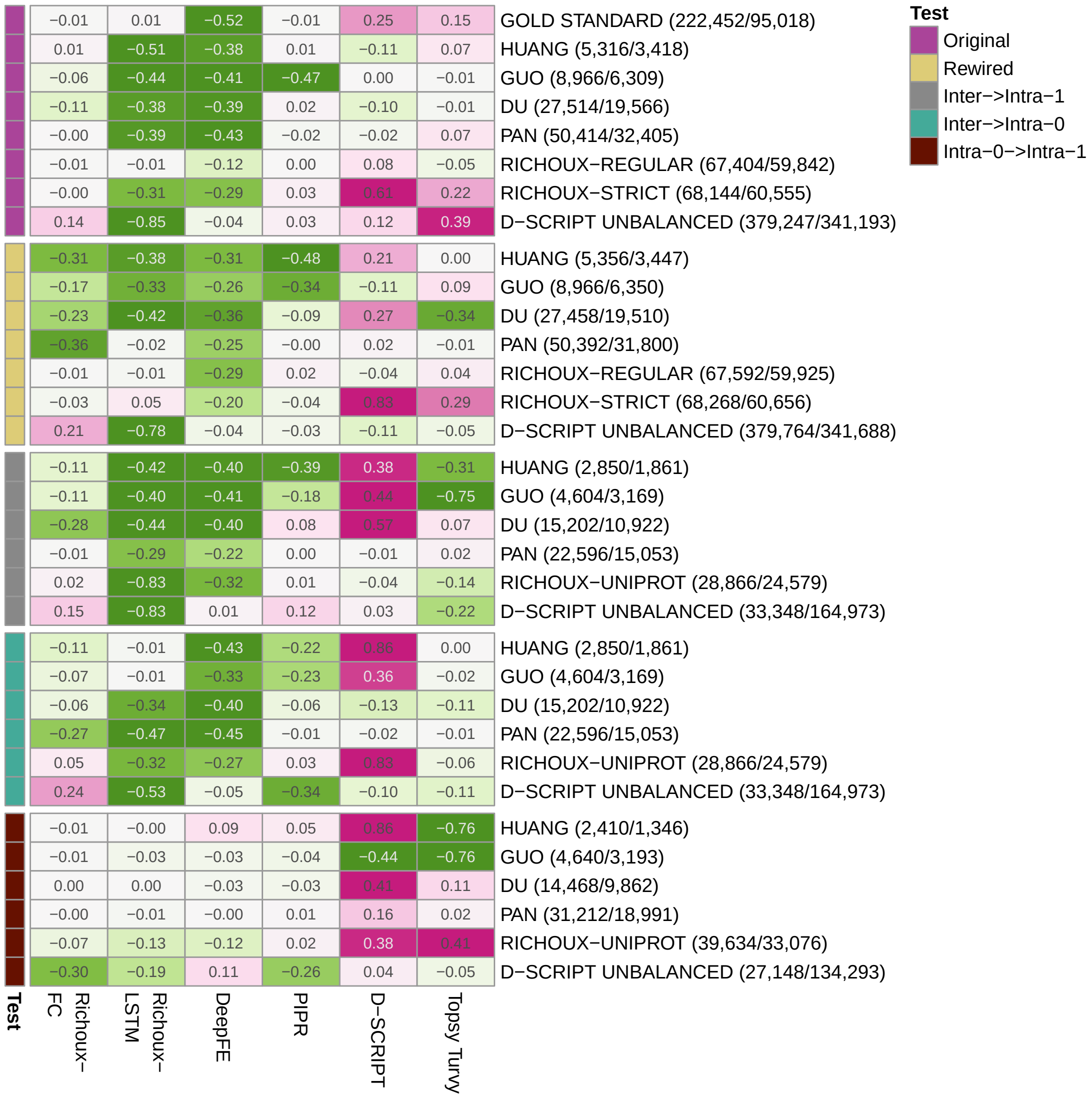

Figure S17: Difference between the precisions from the early stopping setting and the precisions obtained without early stopping. Precision increases especially for D-SCRIPT.

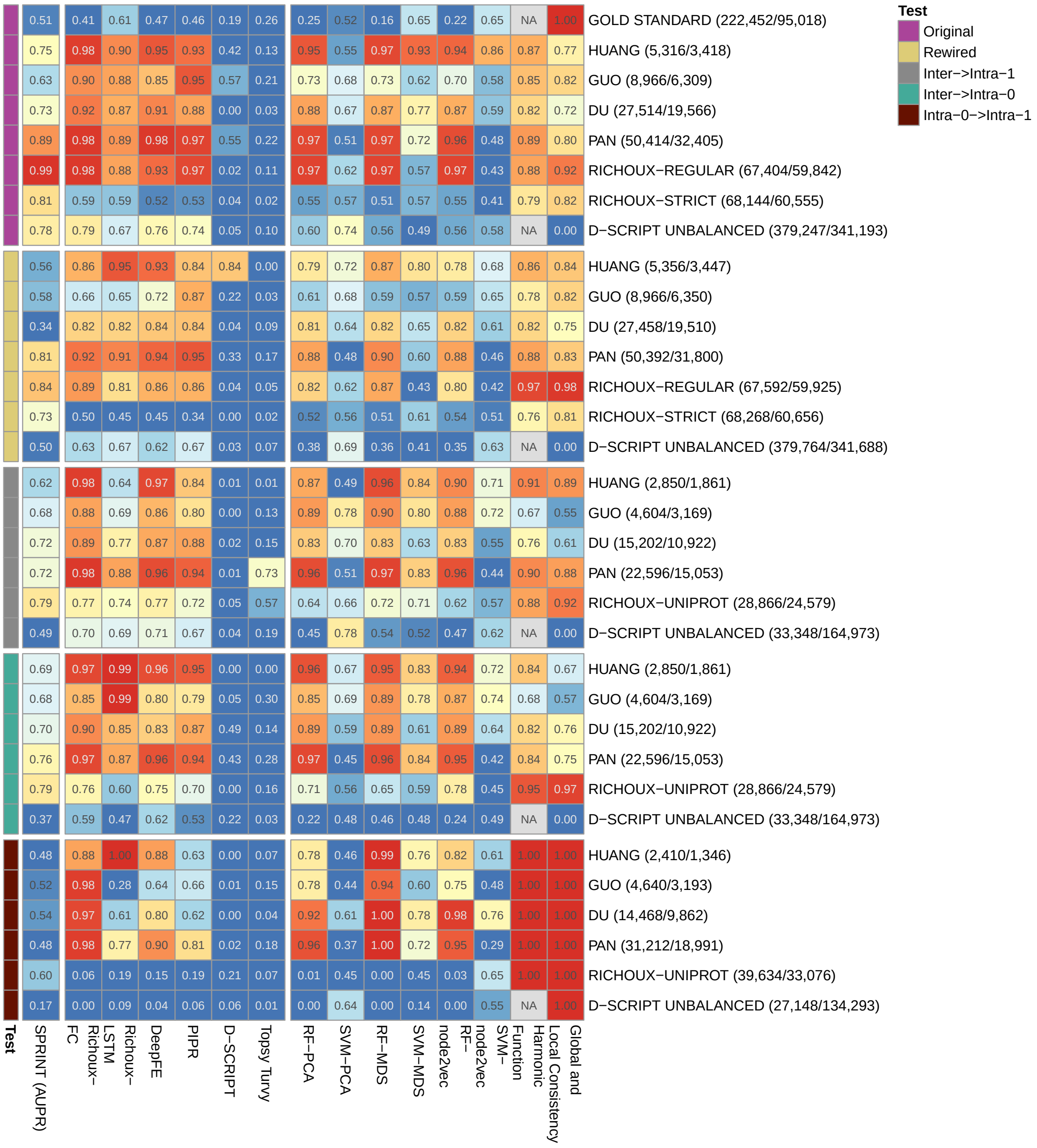

Figure S18: Recall (AUPRs for SPRINT) for all methods and datasets.

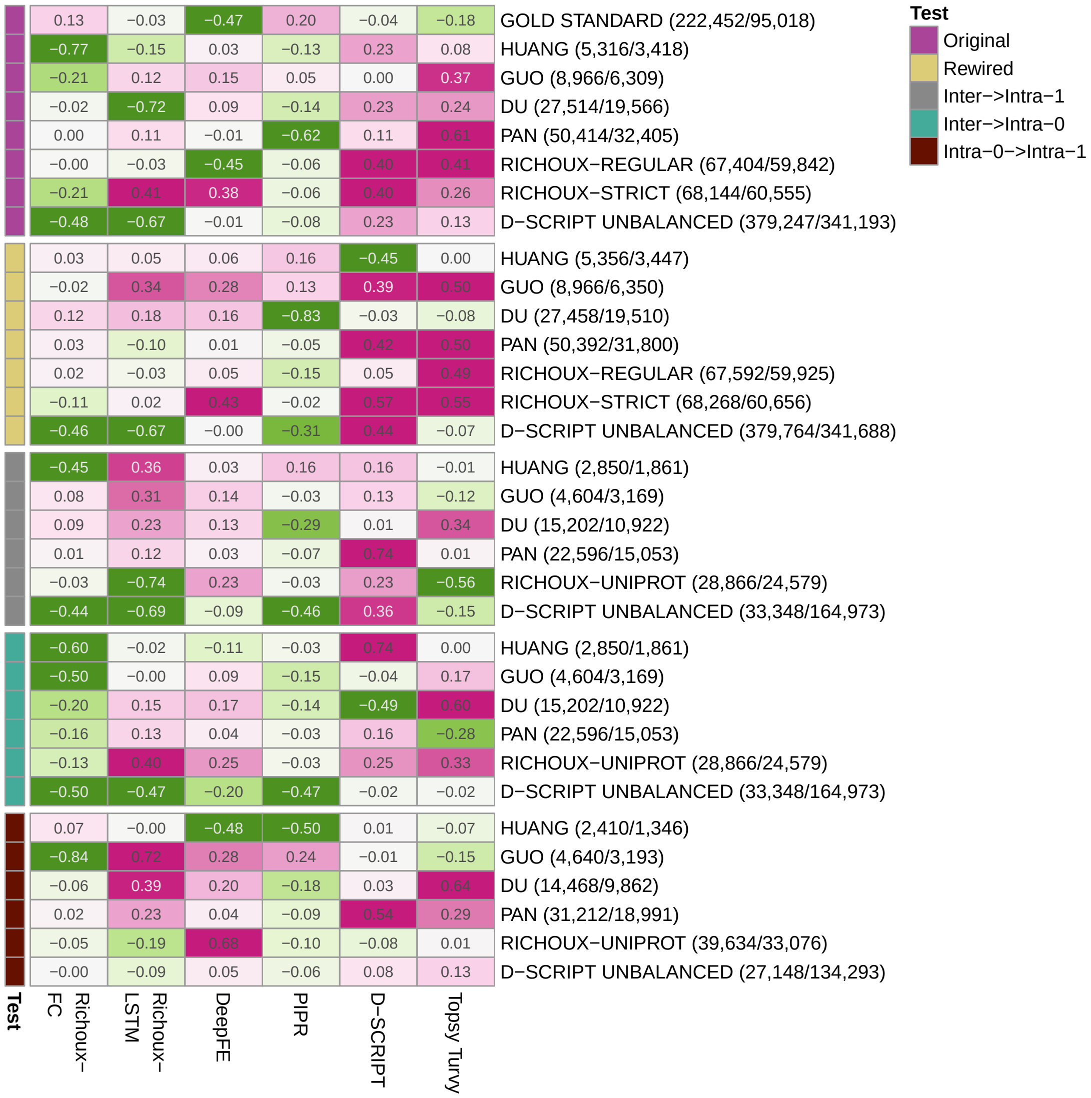

Figure S19: Difference between the recalls from the early stopping setting and the recalls obtained without early stopping. Recall increases for most methods except for Richoux-FC and PIPR.

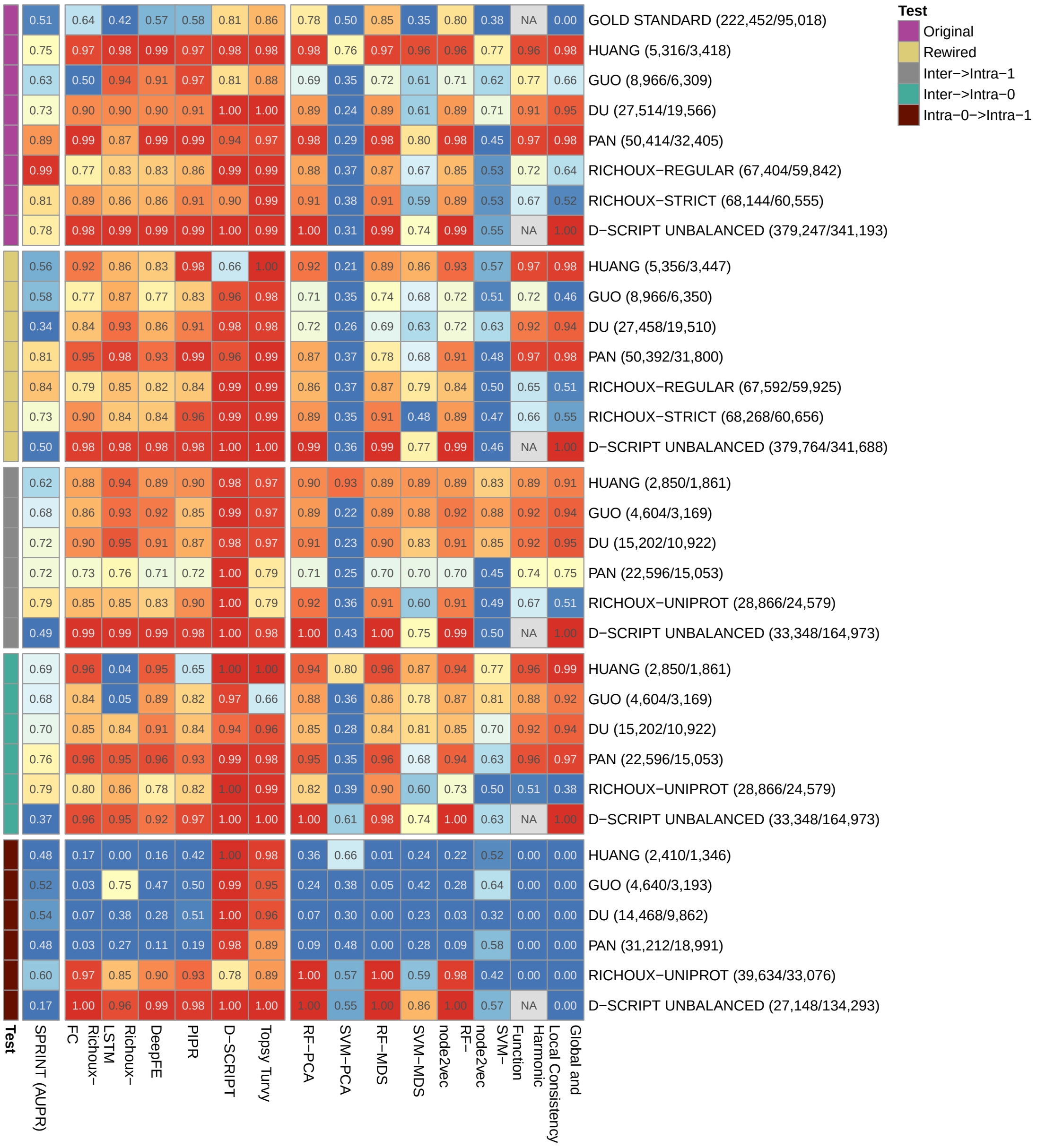

Figure S20: Specificity (AUPRs for SPRINT) for all methods and datasets.

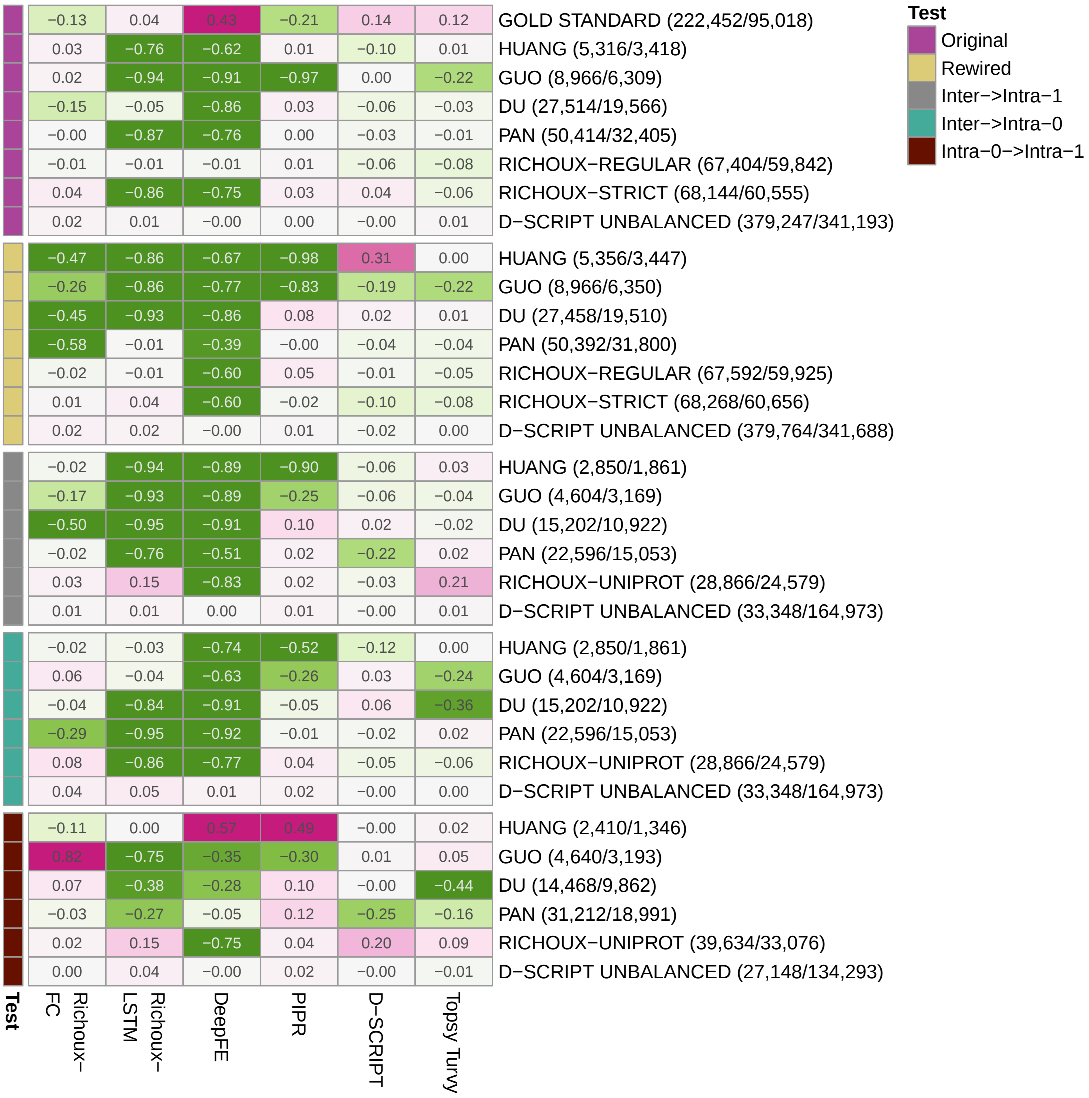

Figure S21: Difference between the specificities from the early stopping setting and the specificities obtained without early stopping. Specificity either mostly decreases (Richoux-LSTM, DeepFE) or stays approximately the same.

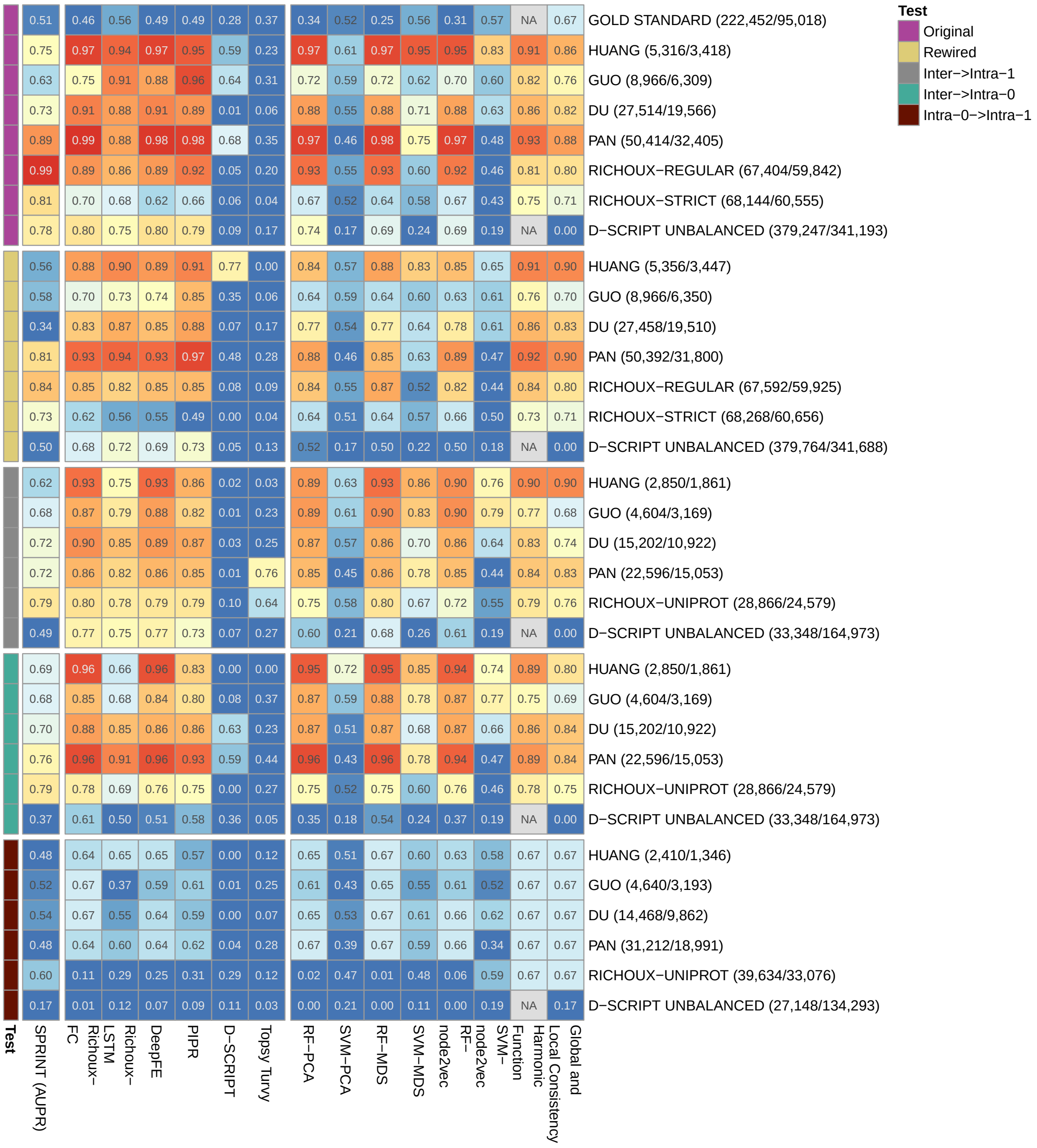

Figure S22: F1 (AUPRs for SPRINT) for all methods and datasets.

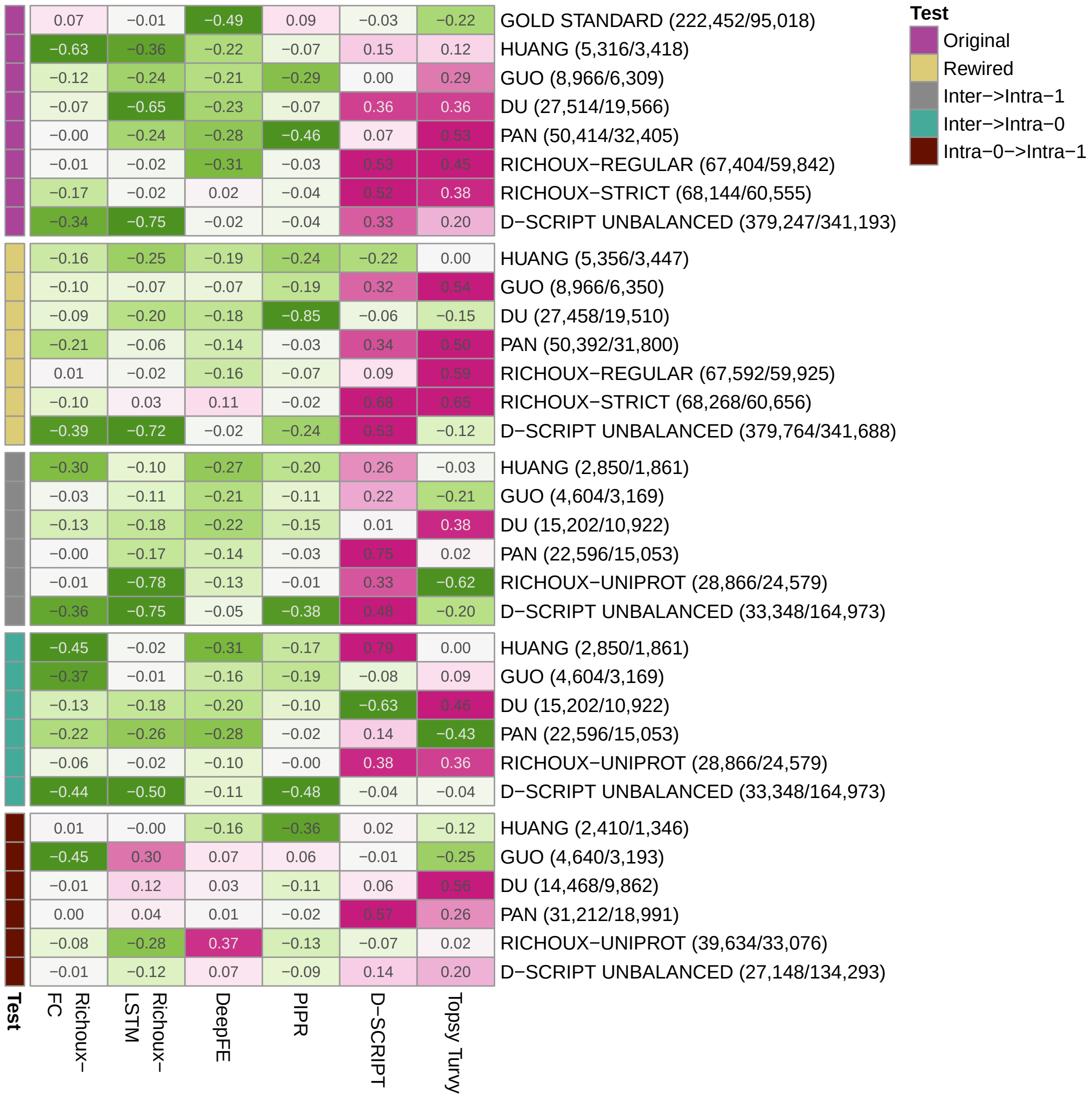

Figure S23: Difference between the F1 scores from the early stopping setting and the F1 scores obtained without early stopping. Mostly D-SCRIPT and Topsy-Turvy profit from early stopping. The performance from Richoux-LSTM and DeepFE improves for the $INTRA_0 \rightarrow INTRA_1$ setting.

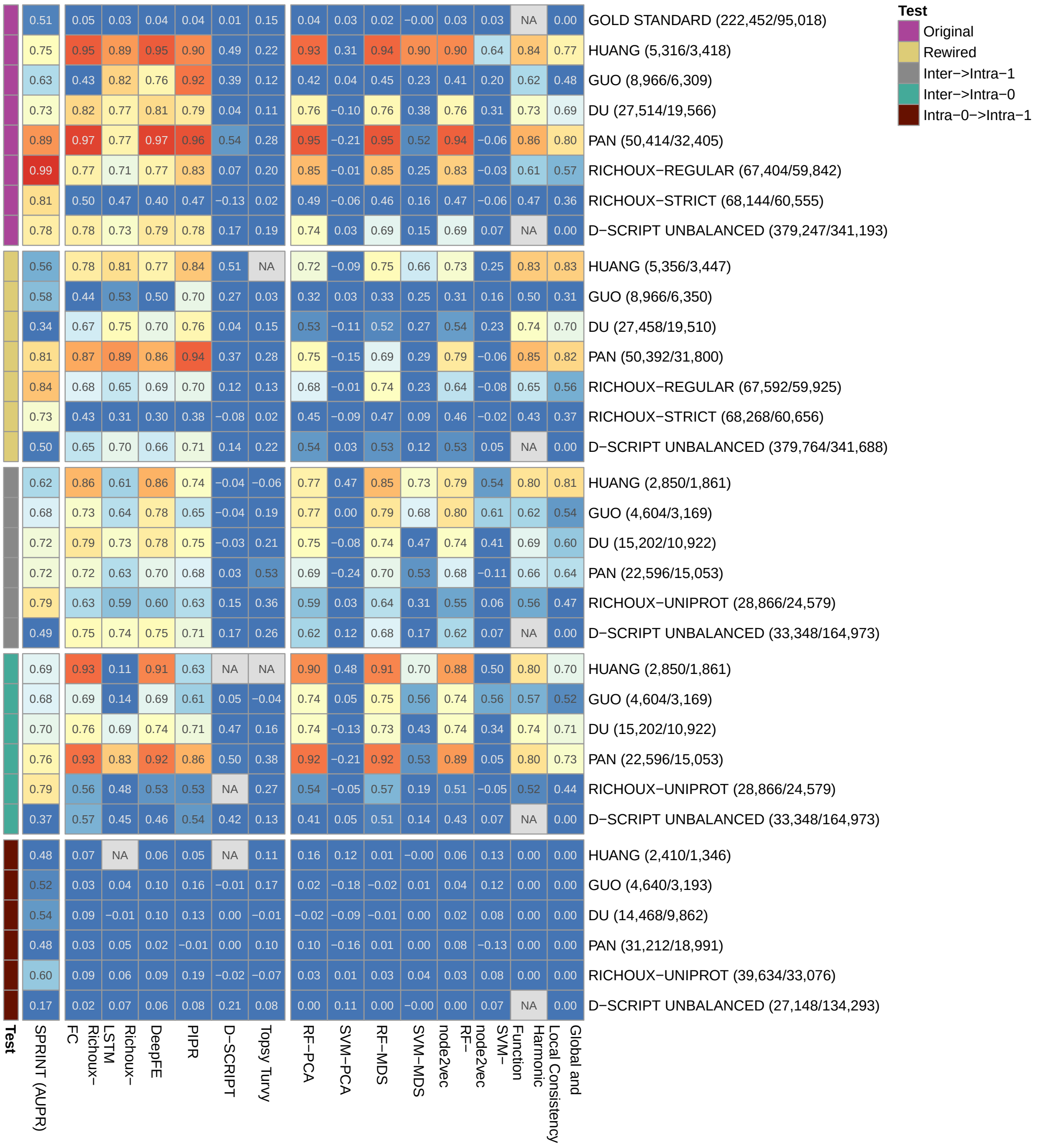

Figure S24: MCC (AUPRs for SPRINT) for all methods and datasets.

Test	Dataset	Richoux-FC	Richoux-LSTM	DeepFE	PIPR	D-SCRIPT	Topsy Turvy
Original	GOLD STANDARD (222,452/95,018)	0.00	0.01	NA	−0.00	0.16	−0.01
Original	HUANG (5,316/3,418)	−0.61	−0.93	−0.50	−0.10	0.06	0.10
Original	GUO (8,966/6,309)	−0.23	NA	NA	NA	0.00	0.12
Original	DU (27,514/19,566)	−0.16	−0.76	−0.68	−0.10	0.22	0.22
Original	PAN (50,414/32,405)	−0.00	NA	−0.67	−0.51	0.06	0.50
Original	RICHOUX−REGULAR (67,404/59,842)	−0.02	−0.04	−0.45	−0.06	0.35	0.26
Original	RICHOUX−STRICT (68,144/60,555)	−0.13	NA	−0.39	−0.01	0.56	0.26
Original	D−SCRIPT UNBALANCED (379,247/341,193)	−0.26	NA	−0.03	−0.03	0.29	0.24
Rewired	HUANG (5,356/3,447)	−0.41	NA	−0.50	NA	−0.07	NA
Rewired	GUO (8,966/6,350)	−0.28	−0.56	NA	NA	0.13	0.27
Rewired	DU (27,458/19,510)	−0.27	NA	−0.68	−0.70	0.00	−0.16
Rewired	PAN (50,392/31,800)	−0.48	−0.10	−0.33	−0.05	0.31	0.37
Rewired	RICHOUX−REGULAR (67,592/59,925)	0.01	−0.04	−0.50	−0.09	0.05	0.41
Rewired	RICHOUX−STRICT (68,268/60,656)	−0.09	0.07	−0.17	−0.04	0.56	0.49
Rewired	D−SCRIPT UNBALANCED (379,764/341,688)	−0.27	NA	−0.02	−0.21	0.42	−0.17
Inter−>Intra−1	HUANG (2,850/1,861)	−0.45	NA	−0.82	NA	0.19	NA
Inter−>Intra−1	GUO (4,604/3,169)	−0.07	NA	−0.67	−0.27	0.15	−0.36
Inter−>Intra−1	DU (15,202/10,922)	−0.32	NA	NA	−0.14	0.13	0.28
Inter−>Intra−1	PAN (22,596/15,053)	0.00	NA	−0.38	−0.06	0.49	0.04
Inter−>Intra−1	RICHOUX−UNIPROT (28,866/24,579)	0.00	NA	NA	−0.01	0.19	−0.35
Inter−>Intra−1	D−SCRIPT UNBALANCED (33,348/164,973)	−0.26	NA	−0.05	−0.29	0.41	−0.19
Inter−>Intra−0	HUANG (2,850/1,861)	−0.56	−0.20	−0.83	−0.56	NA	NA
Inter−>Intra−0	GUO (4,604/3,169)	−0.40	−0.11	−0.50	−0.41	−0.04	−0.06
Inter−>Intra−0	DU (15,202/10,922)	−0.23	NA	NA	−0.18	−0.46	0.17
Inter−>Intra−0	PAN (22,596/15,053)	−0.45	NA	−0.78	−0.04	0.11	−0.33
Inter−>Intra−0	RICHOUX−UNIPROT (28,866/24,579)	−0.03	NA	−0.50	0.01	NA	0.20
Inter−>Intra−0	D−SCRIPT UNBALANCED (33,348/164,973)	−0.30	NA	−0.12	−0.44	−0.05	−0.07
Intra−0−>Intra−1	HUANG (2,410/1,346)	−0.04	NA	0.09	0.00	NA	NA
Intra−0−>Intra−1	GUO (4,640/3,193)	−0.04	NA	−0.04	−0.02	NA	NA
Intra−0−>Intra−1	DU (14,468/9,862)	−0.01	NA	−0.08	−0.09	0.12	0.21
Intra−0−>Intra−1	PAN (31,212/18,991)	−0.04	NA	−0.01	0.04	0.28	0.11
Intra−0−>Intra−1	RICHOUX−UNIPROT (39,634/33,076)	−0.06	−0.07	−0.11	−0.05	0.22	0.20
Intra−0−>Intra−1	D−SCRIPT UNBALANCED (27,148/134,293)	NA	NA	0.07	NA	0.13	0.16

Figure S25: Difference between the MCC scores from the early stopping setting and the MCC scores obtained without early stopping. Mostly D-SCRIPT and Topsy-Turvy profit from early stopping.

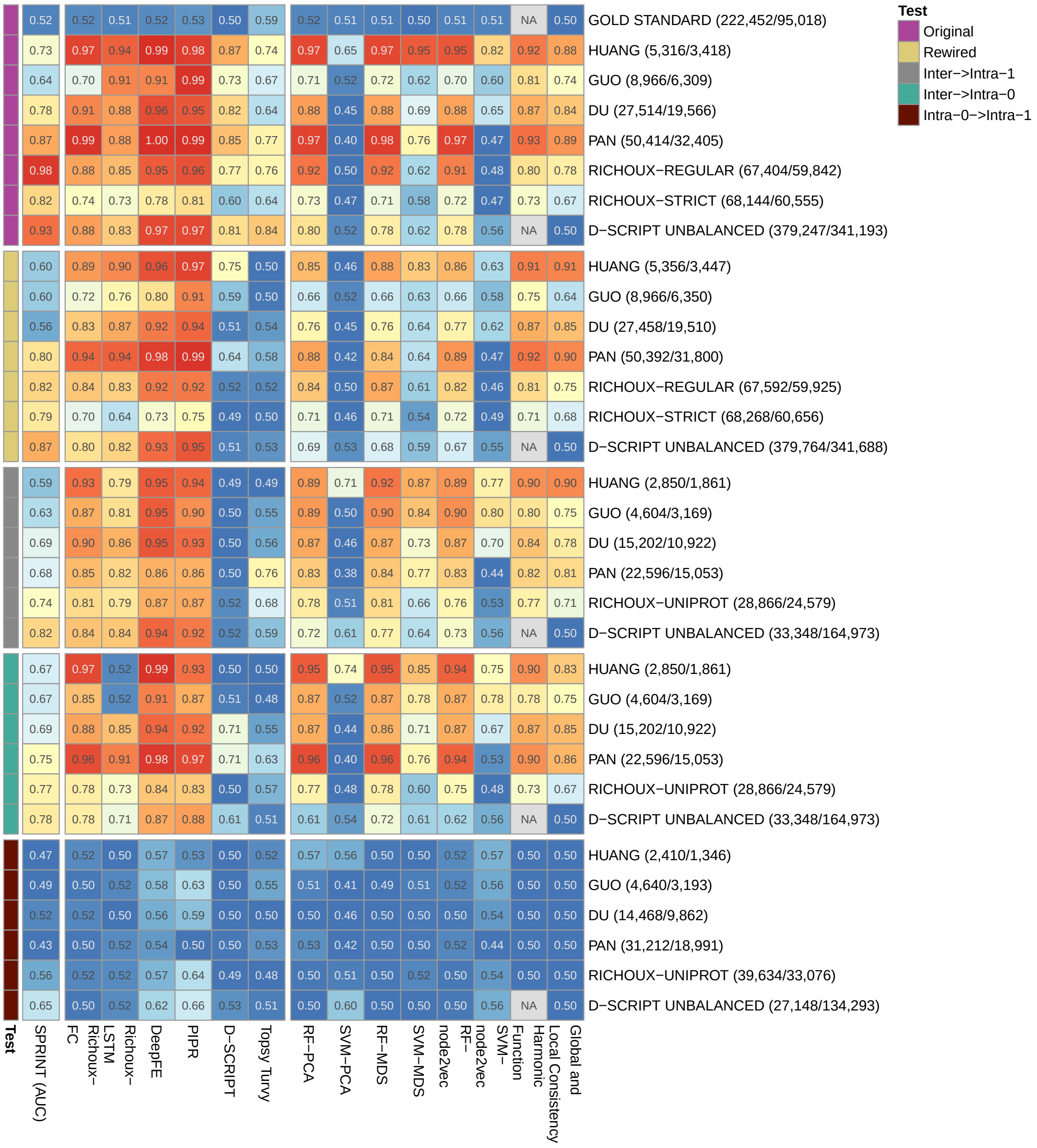

Figure S26: AUC for all methods and datasets.

Test	Richoux−FC	Richoux−LSTM	DeepFE	PIPR	D−SCRIPT	Topsy Turvy	
Original	0.00	0.01	0.01	0.00	0.06	0.02	GOLD STANDARD (222,452/95,018)
Original	−0.37	−0.46	−0.08	−0.01	−0.01	0.14	HUANG (5,316/3,418)
Original	−0.10	−0.41	−0.37	−0.46	0.00	−0.02	GUO (8,966/6,309)
Original	−0.08	−0.38	−0.31	−0.02	−0.06	0.13	DU (27,514/19,566)
Original	−0.00	−0.38	−0.21	−0.12	−0.00	0.19	PAN (50,414/32,405)
Original	−0.01	−0.02	−0.22	−0.01	0.08	0.09	RICHOUX−REGULAR (67,404/59,842)
Original	−0.08	−0.23	−0.20	0.01	0.23	0.09	RICHOUX−STRICT (68,144/60,555)
Original	−0.23	−0.33	−0.01	−0.01	0.05	0.04	D−SCRIPT UNBALANCED (379,247/341,193)
Rewired	−0.22	−0.40	−0.08	−0.40	0.12	−0.11	HUANG (5,356/3,447)
Rewired	−0.14	−0.26	−0.27	−0.39	0.16	0.18	GUO (8,966/6,350)
Rewired	−0.17	−0.37	−0.31	−0.26	0.29	0.04	DU (27,458/19,510)
Rewired	−0.27	−0.06	−0.06	−0.01	0.25	0.27	PAN (50,392/31,800)
Rewired	0.00	−0.02	−0.23	−0.03	0.28	0.34	RICHOUX−REGULAR (67,592/59,925)
Rewired	−0.05	0.03	−0.10	−0.02	0.31	0.32	RICHOUX−STRICT (68,268/60,656)
Rewired	−0.22	−0.32	−0.01	−0.08	0.36	0.38	D−SCRIPT UNBALANCED (379,764/341,688)
Inter−>Intra−1	−0.24	−0.29	−0.33	−0.38	0.22	0.09	HUANG (2,850/1,861)
Inter−>Intra−1	−0.05	−0.31	−0.33	−0.15	0.12	−0.15	GUO (4,604/3,169)
Inter−>Intra−1	−0.20	−0.36	−0.34	−0.01	0.23	0.23	DU (15,202/10,922)
Inter−>Intra−1	−0.00	−0.32	−0.05	0.00	0.33	0.08	PAN (22,596/15,053)
Inter−>Intra−1	−0.00	−0.29	−0.24	−0.01	0.29	0.05	RICHOUX−UNIPROT (28,866/24,579)
Inter−>Intra−1	−0.21	−0.34	−0.02	−0.05	0.36	0.24	D−SCRIPT UNBALANCED (33,348/164,973)
Inter−>Intra−0	−0.31	−0.03	−0.41	−0.32	0.39	−0.10	HUANG (2,850/1,861)
Inter−>Intra−0	−0.22	−0.02	−0.26	−0.23	0.07	−0.05	GUO (4,604/3,169)
Inter−>Intra−0	−0.12	−0.35	−0.34	−0.09	−0.14	0.16	DU (15,202/10,922)
Inter−>Intra−0	−0.22	−0.41	−0.21	−0.01	0.21	0.20	PAN (22,596/15,053)
Inter−>Intra−0	−0.03	−0.23	−0.20	0.01	0.29	0.28	RICHOUX−UNIPROT (28,866/24,579)
Inter−>Intra−0	−0.23	−0.21	−0.05	−0.21	0.22	0.28	D−SCRIPT UNBALANCED (33,348/164,973)
Intra−0−>Intra−1	−0.02	−0.00	0.02	−0.01	0.16	0.03	HUANG (2,410/1,346)
Intra−0−>Intra−1	−0.01	−0.02	−0.04	−0.04	−0.03	−0.12	GUO (4,640/3,193)
Intra−0−>Intra−1	0.01	0.00	−0.02	−0.05	0.01	0.12	DU (14,468/9,862)
Intra−0−>Intra−1	−0.01	−0.02	0.05	0.03	0.18	0.12	PAN (31,212/18,991)
Intra−0−>Intra−1	−0.01	−0.02	−0.09	0.02	0.10	0.01	RICHOUX−UNIPROT (39,634/33,076)
Intra−0−>Intra−1	−0.00	−0.02	0.05	0.09	0.29	0.25	D−SCRIPT UNBALANCED (27,148/134,293)

Test: Original, Rewired, Inter−>Intra−1, Inter−>Intra−0, Intra−0−>Intra−1

Figure S27: Difference between the AUC scores from the early stopping setting and the AUC scores obtained without early stopping. Mostly D-SCRIPT and Topsy-Turvy profit from early stopping.

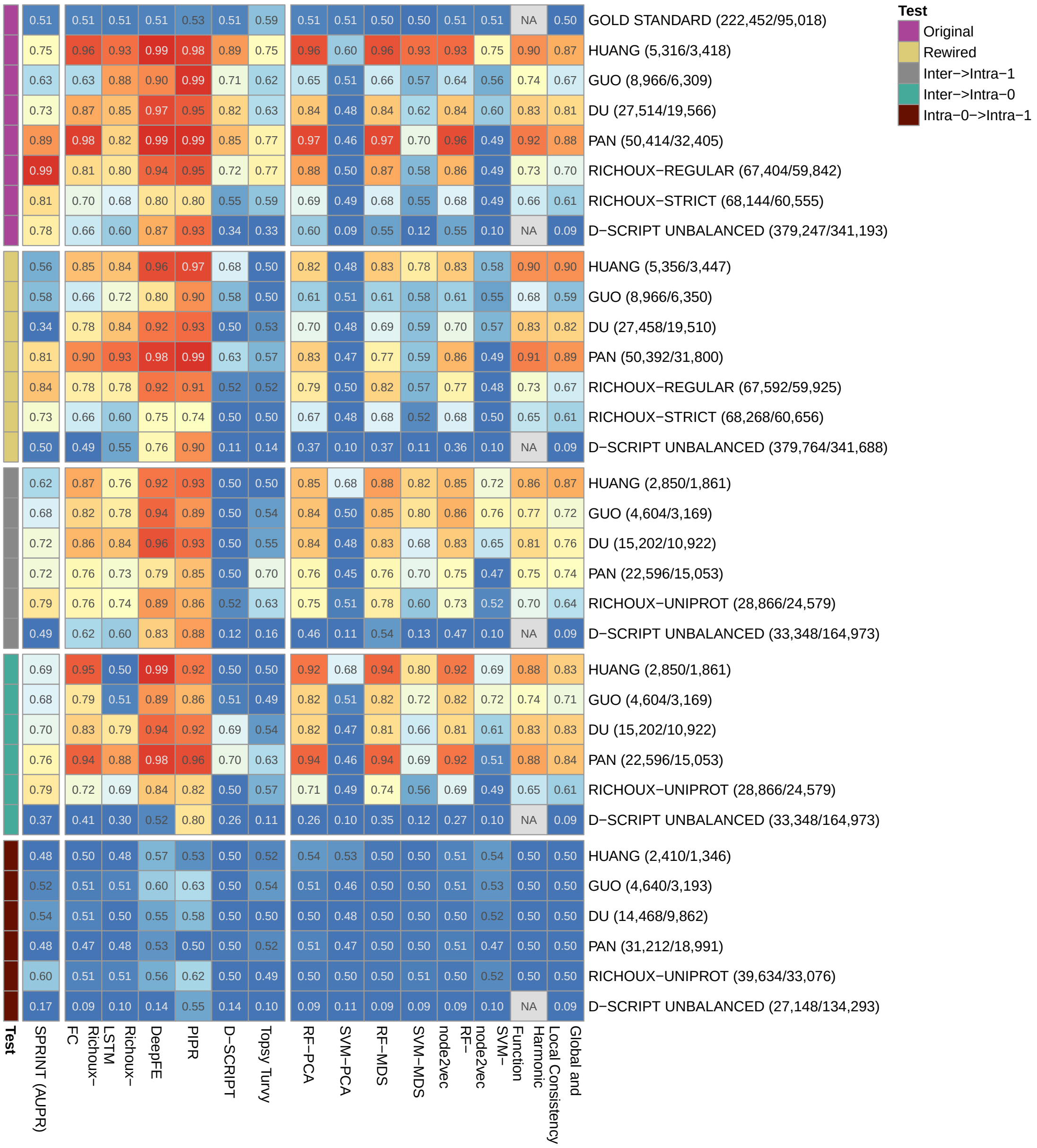

Figure S28: AUPR for all methods and datasets.

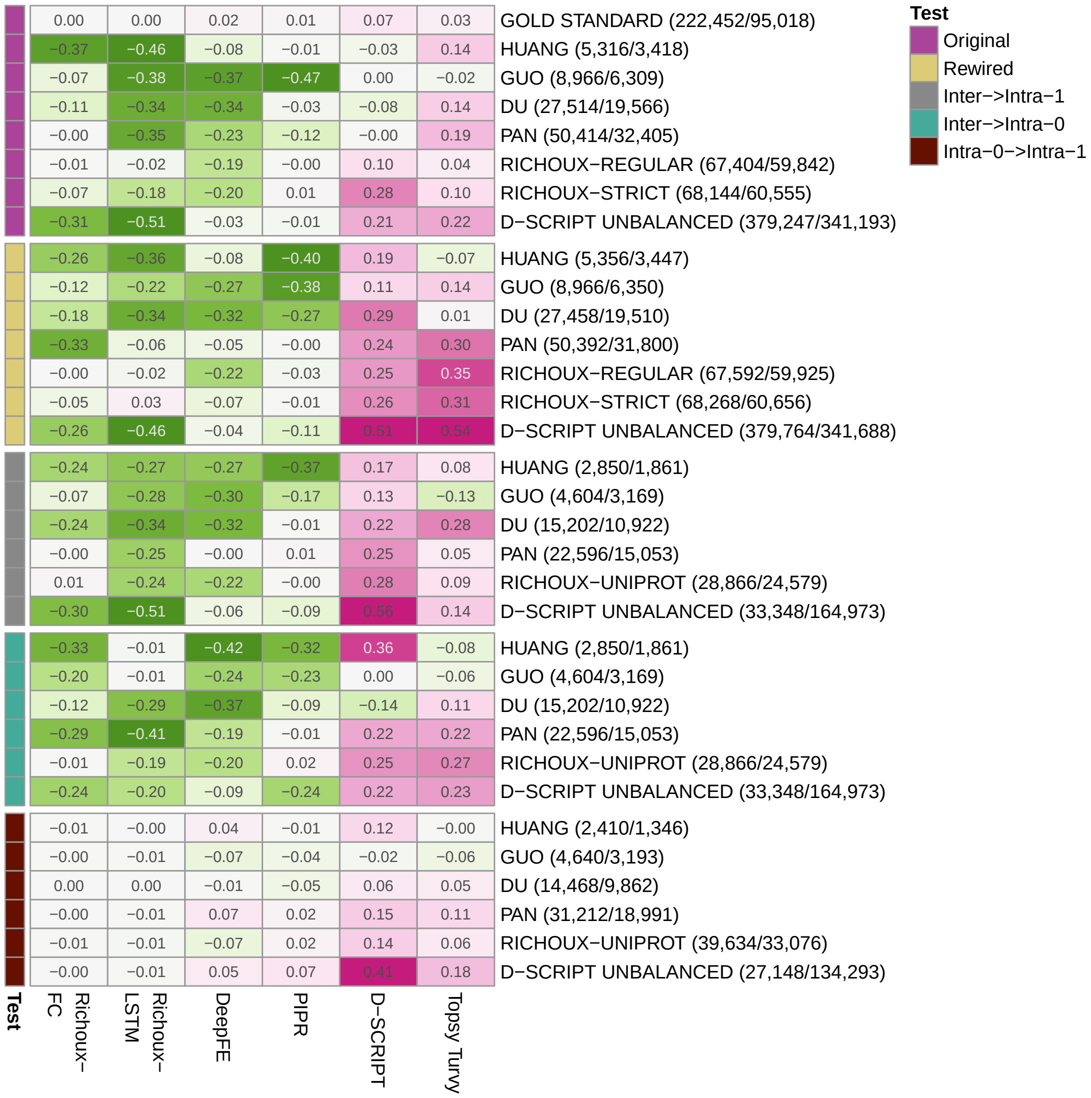

Figure S29: Difference between the AUPR scores from the early stopping setting and the AUPR scores obtained without early stopping. Mostly D-SCRIPT and Topsy-Turvy profit from early stopping.

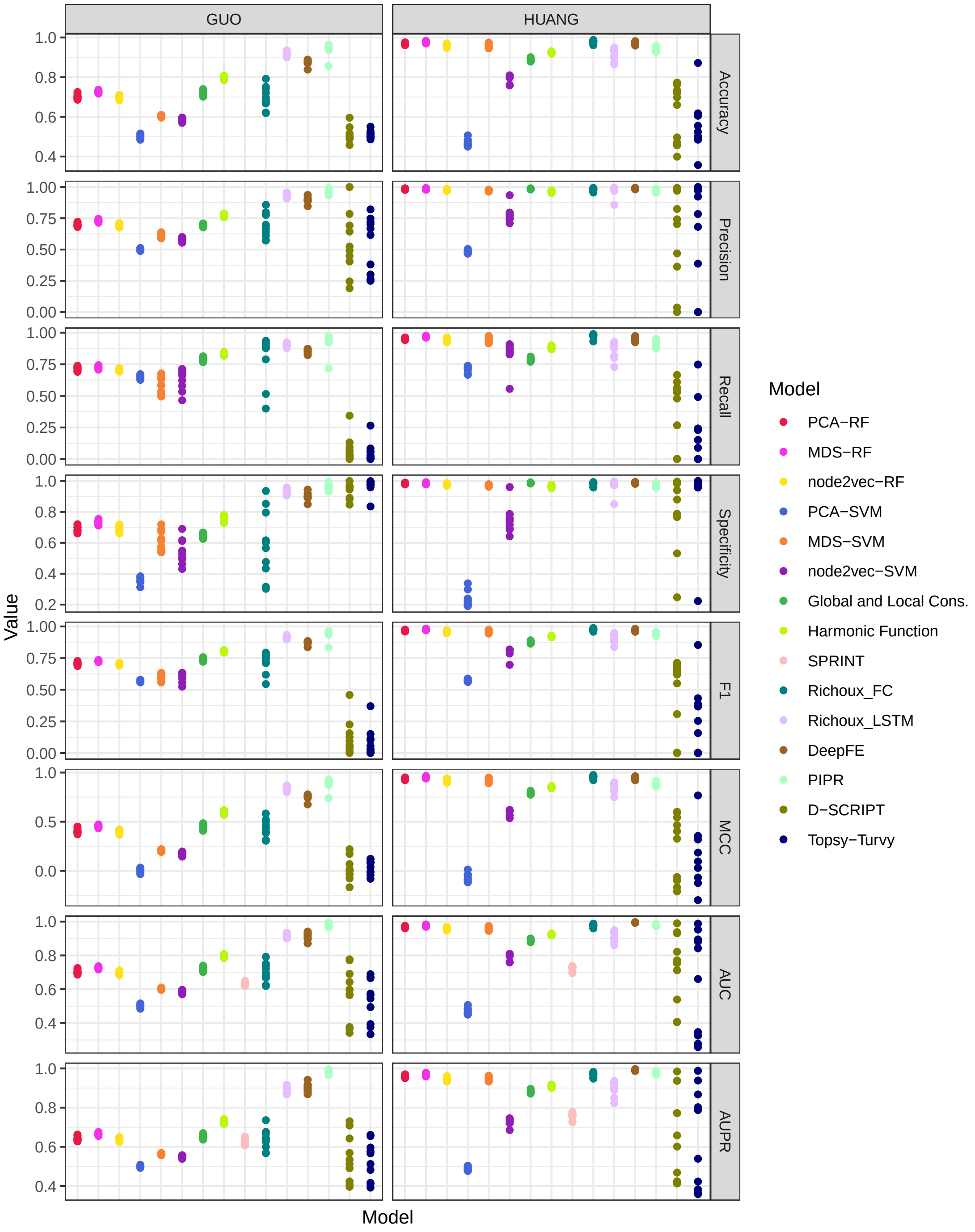

Figure S30: Results of the robustness test on the original datasets. Ten different seeds were used to split the datasets randomly into training (80%) and test (20%). Most methods are robust. Richoux-FC, D-SCRIPT and Topsy-Turvy have the largest variance between different splits.

Model	Dataset	Mean	SD
PIPR	GUO	**0.9408**	0.0315
Richoux-LSTM	GUO	0.9182	0.0111
DeepFE	GUO	0.8749	0.0143
Harmonic Function	GUO	0.7958	0.0076
MDS-RF	GUO	0.7268	0.0060
Global and Local Cons.	GUO	0.7196	0.0103
PCA-RF	GUO	0.7022	0.0120
node2vec-RF	GUO	0.7014	0.0076
Richoux-FC	GUO	0.6965	**0.0556**
MDS-SVM	GUO	0.6040	0.0049
node2vec-SVM	GUO	0.5865	0.0095
Topsy-Turvy	GUO	0.5110	0.0183
D-SCRIPT	GUO	0.5091	0.0378
PCA-SVM	GUO	0.5040	0.0090
MDS-RF	HUANG	**0.9780**	0.0043
Richoux-FC	HUANG	**0.9780**	0.0080
DeepFE	HUANG	0.9712	0.0060
PCA-RF	HUANG	0.9673	0.0048
node2vec-RF	HUANG	0.9610	0.0074
MDS-SVM	HUANG	0.9592	0.0082
PIPR	HUANG	0.9455	0.0094
Harmonic Function	HUANG	0.9240	0.0044
Richoux-LSTM	HUANG	0.9146	0.0296
Global and Local Cons.	HUANG	0.8876	0.0070
node2vec-SVM	HUANG	0.7947	0.0191
D-SCRIPT	HUANG	0.6171	**0.1443**
Topsy-Turvy	HUANG	0.5512	0.1338
PCA-SVM	HUANG	0.4713	0.0171

Table S8: Mean and standard deviation over the ten accuracies obtained on the ten different random splits of the original datasets. Results are ranked by their mean, the best mean performance and largest standard deviation per dataset are indicated in bold.

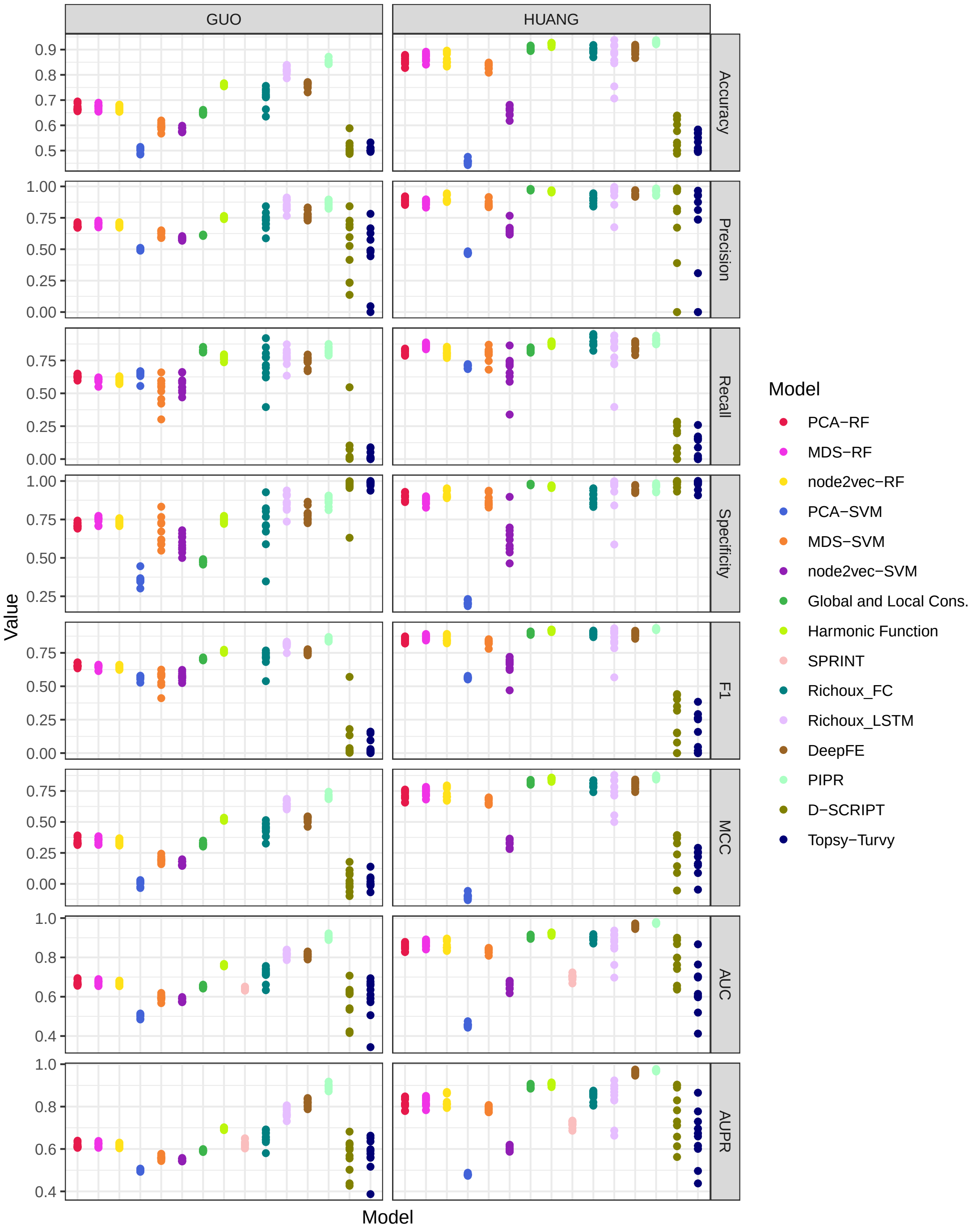

Figure S31: Results of the robustness test on the rewired datasets. Ten different seeds were used to split the datasets randomly into training (80%) and test (20%). Most methods are robust. Richoux-LSTM, Richoux-FC, D-SCRIPT and Topsy-Turvy have the largest variance between different splits.

Model	Dataset	Mean	SD
PIPR	GUO	**0.8515**	0.0094
Richoux-LSTM	GUO	0.8186	0.0160
Harmonic Function	GUO	0.7603	0.0046
DeepFE	GUO	0.7598	0.0127
Richoux-FC	GUO	0.7129	**0.0371**
MDS-RF	GUO	0.6729	0.0110
PCA-RF	GUO	0.6695	0.0126
node2vec-RF	GUO	0.6663	0.0109
Global and Local Cons.	GUO	0.6509	0.0054
MDS-SVM	GUO	0.5952	0.0140
node2vec-SVM	GUO	0.5814	0.0095
D-SCRIPT	GUO	0.5122	0.0295
Topsy-Turvy	GUO	0.5047	0.0109
PCA-SVM	GUO	0.5028	0.0095
PIPR	HUANG	**0.9292**	0.0051
Harmonic Function	HUANG	0.9219	0.0056
Global and Local Cons.	HUANG	0.9059	0.0082
DeepFE	HUANG	0.8972	0.0165
Richoux-FC	HUANG	0.8968	0.0135
MDS-RF	HUANG	0.8682	0.0155
Richoux-LSTM	HUANG	0.8623	**0.0759**
node2vec-RF	HUANG	0.8601	0.0222
PCA-RF	HUANG	0.8544	0.0186
MDS-SVM	HUANG	0.8338	0.0122
node2vec-SVM	HUANG	0.6534	0.0199
D-SCRIPT	HUANG	0.5620	0.0606
Topsy-Turvy	HUANG	0.5394	0.0361
PCA-SVM	HUANG	0.4559	0.0088

Table S9: Mean and standard deviation over the ten accuracies obtained on the ten different random splits of the rewired datasets. Results are ranked by their mean, the best mean performance and largest standard deviation per dataset are indicated in bold.

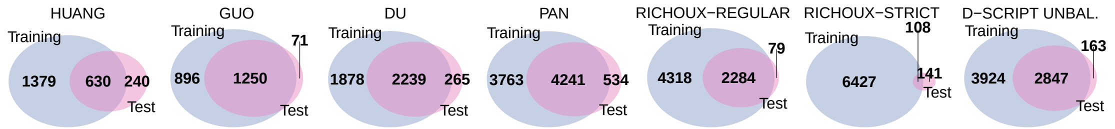

(a) Overlap of proteins occurring in the positive original training and test sets.

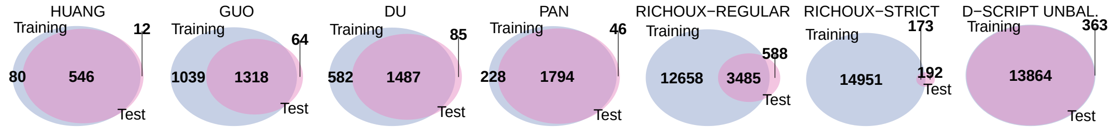

(b) Overlap of proteins occurring in the negative original training and test sets.

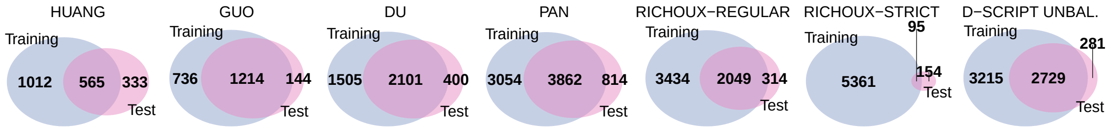

(c) Overlap of proteins occurring in the positive rewired training and test sets.

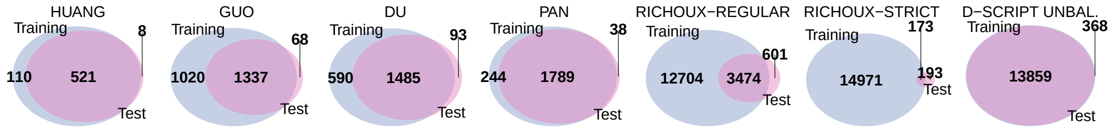

(d) Overlap of proteins occurring in the negative rewired training and test sets.

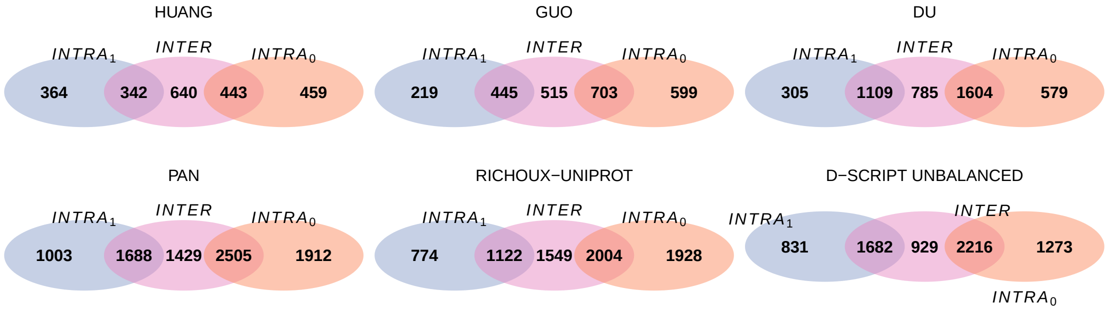

(e) Overlap of proteins occurring in the positive partitioned training and test sets.

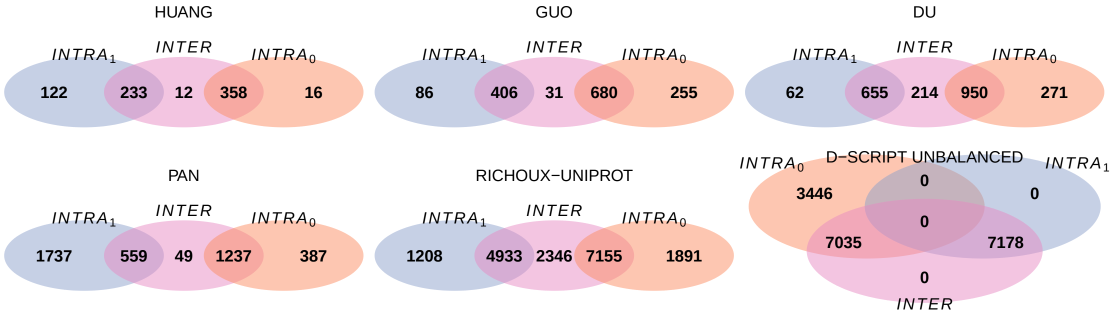

(f) Overlap of proteins occurring in the negative partitioned training and test sets.

Figure S32: Overlap of proteins for all datasets, visualized separately for positive and negative datasets.

References

[1] Richoux, F., Servantie, C., Borès, C. & Téletchéa, S. Comparing two deep learning sequence-based models for protein-protein interaction prediction. *arXiv preprint arXiv:1901.06268* (2019).

[2] Chen, M. *et al.* Multifaceted protein–protein interaction prediction based on siamese residual rcnn. *Bioinformatics* **35**, i305–i314 (2019).

[3] Yao, Y., Du, X., Diao, Y. & Zhu, H. An integration of deep learning with feature embedding for protein–protein interaction prediction. *PeerJ* **7**, e7126 (2019).

[4] Sledzieski, S., Singh, R., Cowen, L. & Berger, B. D-script translates genome to phenome with sequence-based, structure-aware, genome-scale predictions of protein-protein interactions. *Cell Systems* **12**, 969–982 (2021).

[5] Singh, R., Devkota, K., Sledzieski, S., Berger, B. & Cowen, L. Topsy-turvy: integrating a global view into sequence-based ppi prediction. *Bioinformatics* **38**, i264–i272 (2022).

[6] Nambiar, A. *et al.* Transforming the language of life: transformer neural networks for protein prediction tasks. In *Proceedings of the 11th ACM international conference on bioinformatics, computational biology and health informatics*, 1–8 (2020).

[7] Ieremie, I., Ewing, R. M. & Niranjan, M. Transformergo: predicting protein–protein interactions by modelling the attention between sets of gene ontology terms. *Bioinformatics* **38**, 2269–2277 (2022).

[8] You, Z.-H., Chan, K. C. & Hu, P. Predicting protein-protein interactions from primary protein sequences using a novel multi-scale local feature representation scheme and the random forest. *PloS one* **10**, e0125811 (2015).

[9] Ding, Y., Tang, J. & Guo, F. Predicting protein-protein interactions via multivariate mutual information of protein sequences. *BMC bioinformatics* **17**, 1–13 (2016).

[10] Hashemifar, S., Neyshabur, B., Khan, A. A. & Xu, J. Predicting protein–protein interactions through sequence-based deep learning. *Bioinformatics* **34**, i802–i810 (2018).

[11] Hamp, T. & Rost, B. Evolutionary profiles improve protein–protein interaction prediction from sequence. *Bioinformatics* **31**, 1945–1950 (2015).

[12] Li, Y. & Ilie, L. Sprint: ultrafast protein-protein interaction prediction of the entire human interactome. *BMC bioinformatics* **18**, 1–11 (2017).

[13] Bepler, T. & Berger, B. Learning protein sequence embeddings using information from structure. *arXiv preprint arXiv:1902.08661* (2019).

[14] Arnold, R., Goldenberg, F., Mewes, H.-W. & Rattei, T. Simap—the database of all-against-all protein sequence similarities and annotations with new interfaces and increased coverage. *Nucleic acids research* **42**, D279–D284 (2014).

[15] Zhu, X., Ghahramani, Z. & Lafferty, J. D. Semi-supervised learning using gaussian fields and harmonic functions. In *Proceedings of the 20th International conference on Machine learning (ICML-03)*, 912–919 (2003).

[16] Zhou, D., Bousquet, O., Lal, T., Weston, J. & Schölkopf, B. Learning with local and global consistency. *Advances in neural information processing systems* **16** (2003).

[17] Guo, Y., Yu, L., Wen, Z. & Li, M. Using support vector machine combined with auto covariance to predict protein–protein interactions from protein sequences. *Nucleic acids research* **36**, 3025–3030 (2008).

[18] Guo, Y. *et al.* Pred_ppi: a server for predicting protein-protein interactions based on sequence data with probability assignment. *BMC research notes* **3**, 1–7 (2010).

[19] Pan, X.-Y., Zhang, Y.-N. & Shen, H.-B. Large-scale prediction of human protein- protein interactions from amino acid sequence based on latent topic features. *Journal of proteome research* **9**, 4992–5001 (2010).

[20] Sun, T., Zhou, B., Lai, L. & Pei, J. Sequence-based prediction of protein protein interaction using a deep-learning algorithm. *BMC bioinformatics* **18**, 1–8 (2017).

[21] Wang, L., Wang, H.-F., Liu, S.-R., Yan, X. & Song, K.-J. Predicting protein-protein interactions from matrix-based protein sequence using convolution neural network and feature-selective rotation forest. *Scientific reports* **9**, 1–12 (2019).

[22] Xu, D., Xu, H., Zhang, Y., Chen, W. & Gao, R. Protein-protein interactions prediction based on graph energy and protein sequence information. *Molecules* **25**, 1841 (2020).

[23] Wang, J., Zhang, L., Jia, L., Ren, Y. & Yu, G. Protein-protein interactions prediction using a novel local conjoint triad descriptor of amino acid sequences. *International Journal of Molecular Sciences* **18**, 2373 (2017).

[24] Hu, L. & Chan, K. C. Discovering variable-length patterns in protein sequences for protein-protein interaction prediction. *IEEE transactions on nanobioscience* **14**, 409–416 (2015).

[25] Du, X. *et al.* Deepppi: boosting prediction of protein–protein interactions with deep neural networks. *Journal of chemical information and modeling* **57**, 1499–1510 (2017).

[26] Jha, K. & Saha, S. Amalgamation of 3d structure and sequence information for protein–protein interaction prediction. *Scientific Reports* **10**, 1–14 (2020).

[27] Saha, I. *et al.* Ensemble learning prediction of protein–protein interactions using proteins functional annotations. *Molecular BioSystems* **10**, 820–830 (2014).

[28] Chen, K.-H., Wang, T.-F. & Hu, Y.-J. Protein-protein interaction prediction using a hybrid feature representation and a stacked generalization scheme. *BMC bioinformatics* **20**, 1–17 (2019).

[29] Zhao, L., Wang, J., Hu, Y. & Cheng, L. Conjoint feature representation of go and protein sequence for ppi prediction based on an inception rnn attention network. *Molecular Therapy-Nucleic Acids* **22**, 198–208 (2020).

[30] Maetschke, S. R., Simonsen, M., Davis, M. J. & Ragan, M. A. Gene ontology-driven inference of protein–protein interactions using inducers. *Bioinformatics* **28**, 69–75 (2012).

[31] Shen, J. *et al.* Predicting protein–protein interactions based only on sequences information. *Proceedings of the National Academy of Sciences* **104**, 4337–4341 (2007).

[32] Mahapatra, S., Kumar, A., Sharma, A. & Sahu, S. S. Effect of dimensionality reduction on classification accuracy for protein–protein interaction prediction. In *Advanced Computing and Intelligent Engineering*, 3–12 (Springer, 2020).

[33] Wang, L. *et al.* Using two-dimensional principal component analysis and rotation forest for prediction of protein-protein interactions. *Scientific reports* **8**, 1–10 (2018).

[34] Pazos, F. & Valencia, A. Similarity of phylogenetic trees as indicator of protein–protein interaction. *Protein engineering* **14**, 609–614 (2001).

[35] Ochoa, D., Juan, D., Valencia, A. & Pazos, F. Detection of significant protein coevolution. *Bioinformatics* **31**, 2166–2173 (2015).

[36] Humphreys, I. R. *et al.* Computed structures of core eukaryotic protein complexes. *Science* **374**, eabm4805 (2021).
